# Arsenite-induced stress granule formation is inhibited by elevated levels of reduced glutathione in West Nile virus-infected cells

**DOI:** 10.1371/journal.ppat.1006240

**Published:** 2017-02-27

**Authors:** Mausumi Basu, Sean C. Courtney, Margo A. Brinton

**Affiliations:** Department of Biology, Georgia State University, Atlanta, GA, United States of America; The Scripps Research Institute, UNITED STATES

## Abstract

Oxidative stress activates the cellular kinase HRI, which then phosphorylates eIF2α, resulting in stalled translation initiation and the formation of stress granules (SGs). SG assembly redirects cellular translation to stress response mRNAs and inhibits cap-dependent viral RNA translation. Flavivirus infections were previously reported to induce oxidative stress in infected cells but flavivirus-infected cells paradoxically develop resistance to arsenite (Ars)-induced SG formation with time after infection. This resistance was previously postulated to be due to sequestration of the SG protein Caprin1 by Japanese encephalitis virus capsid protein. However, Caprin1 did not co-localize with West Nile virus (WNV) capsid protein in infected cells. Other stressors induced SGs with equal efficiency in mock- and WNV-infected cells indicating the intrinsic ability of cells to assemble SGs was not disabled. Induction of both reactive oxygen species (ROS) and the antioxidant response was detected at early times after WNV-infection. The transcription factors, Nrf2 and ATF4, which activate antioxidant genes, were upregulated and translocated to the nucleus. Knockdown of Nrf2, ATF4 or apoptosis-inducing factor (AIF), a mitochondrial protein involved in regenerating intracellular reduced glutathione (GSH) levels, with siRNA or treatment of cells with buthionine sulphoximine, which induces oxidative stress by inhibiting GSH synthesis, decreased intracellular GSH levels and increased the number of SG-positive, infected cells. Mitochondria were protected from Ars-induced damage by WNV infection until late times in the infection cycle. The results indicate that the increase in virus-induced ROS levels is counterbalanced by a virus-induced antioxidant response that is sufficient to also overcome the increase in ROS induced by Ars treatment and prevent Ars-induced SG assembly and mitochondrial damage. The virus-induced alterations in the cellular redox status appear to provide benefits for the virus during its lifecycle.

## Author summary

West Nile virus (WNV) was introduced into the United States in 1999 and has since become the major cause of arboviral encephalitis. How a WNV infection manipulates/utilizes cell stress responses is not well understood and gaining a greater understanding may reveal novel targets for the development of antiviral therapies. Even though infections with WNV and other flaviviruses induce increased levels of reactive oxygen species (ROS) typically associated with oxidative stress, infected cells do not display characteristic effects of this stress, such as stalled mRNA translation initiation, stress granule (SG) assembly and mitochondrial damage. Arsenite-treatment of uninfected cells induces high levels of ROS, but flavivirus-infected cells are resistant to arsenite-induced oxidative stress. The mechanisms controlling this resistance were investigated. We first showed that WNV-infected cells are fully susceptible to other types of exogenous stresses that induce SGs. This indicated that virus infection does not disable SG assembly. We then found that cellular antioxidant responses are highly upregulated by virus infection and that the capacity of the antioxidant response is sufficient to counterbalance the negative effects of both virus- and arsenite-induced ROS. The upregulation of both cellular oxidative and antioxidant responses appears to provide advantages for virus replication in cells.

## Introduction

West Nile virus (WNV) is a member of the genus *Flavivirus* within the family *Flaviviridae* that also includes other important human pathogens, such as dengue virus (DENV), yellow fever virus, Zika virus, Japanese encephalitis virus (JEV) and tick-borne encephalitis virus [1]. The positive-sense, single-stranded WNV RNA genome is about 11 kb in length and encodes a single polyprotein that is cleaved by both viral and host cell proteases to produce three structural (E, prM/M, and C) and seven nonstructural (NS1, NS2A, NS2B, NS3, NS4A, NS4B, and NS5) proteins [1]. WNV is maintained in nature in a mosquito-bird transmission cycle. Since its initial isolation in Uganda in 1937, WNV has spread globally and is now endemic in Africa, the Middle East, West Asia, Australia, and since 1999, in the Americas. The majority of WNV infections in humans are asymptomatic but about 20% develop mild flu-like symptoms and about 1% develop neuroinvasive disease that can be fatal [2–4]. Over the last 10 years, WNV has become the leading cause of mosquito-borne encephalitis in the United States. Infections in more than 42,000 people were reported to CDC between 1999 and 2014 with 18,810 individuals displaying neuroinvasive disease and more than 1,700 fatal cases.

In response to many types of stress, cells respond by downregulating global translation. This is usually accomplished by phosphorylation of eukaryotic translation initiation factor 2α (eIF2α) [5] by one of four kinases, protein kinase R (PKR), PKR-like endoplasmic reticulum (ER) kinase (PERK), heme-regulated inhibitor kinase (HRI), or general control non-repressed 2 kinase (GCN2) [6]. PKR is activated by double-stranded RNA in virus-infected cells, PERK is activated by the accumulation of unfolded proteins in the endoplasmic reticulum (ER), HRI is activated by increased levels of reactive oxygen species (ROS), and GCN2 is activated by amino acid deprivation. Phosphorylation of eIF2α prevents recycling of the ternary tRNA^Met^-GTP-eIF2 complex resulting in stalled translation initiation complexes that are assembled into discrete cytoplasmic foci known as stress granules (SGs). SGs contain translationally repressed mRNAs, translation initiation factors, the small ribosomal subunit, and SG-nucleating, RNA-binding proteins, as well as many other types of proteins [7]. We previously reported that infections with natural strains of WNV do not induce the formation of SGs in BHK cells because PKR is not activated [8]. At early times after infection, WNV RNA levels are kept low and the viral RNA is membrane-associated, and at later times, exponential viral RNA replication takes place within virus-induced invaginations in the ER membrane.

Incubation of cells with Ars induces the production of reactive oxygen species (ROS) that activate HRI to phosphorylate eIF2α and initiate SG formation [9]. ROS include free radicals, such as superoxide anion and hydroxyl radicals, as well as non-radical molecules, including hydrogen peroxide (H_2_O_2_) and singlet oxygen. In cells, ROS levels are regulated by both the rate of reduction of O_2_ to superoxide (O_2_^.−^) by the mitochondrial electron transport chain and by the rate of electron scavenging mediated by antioxidant pathway products. When ROS levels are increased, oxidative stress is induced. Electron scavenging by antioxidant pathway products, which donate electrons to ROS, reduces ROS levels and oxidative stress [10–12]. Reduced glutathione (GSH) functions as the main cellular electron scavenger. Cellular GSH levels are regulated by both the rates of its synthesis and of its regeneration from oxidized glutathione (GSSG) by antioxidant enzymes. The expression of antioxidant pathway genes involved in GSH synthesis and regeneration is activated by the transcription factors, activating transcription factor 4 (ATF4) and NF-E2-related factor 2 (Nrf2) [13–17]. Apoptosis inducing factor (AIF) is an FAD-dependent flavoenzyme located in the mitochondrial intermembrane space of mammalian cells that acts as an NAD(P)H-dependent oxidoreductase to regenerate GSH [18].

Infections of cells with several different flaviviruses were previously reported to induce ROS production [19–22]. Paradoxically, WNV-, DENV-, and JEV-infected cells develop resistance to Ars-induced oxidative stress and SG formation [23–25]. A previous study reported that inhibition of Ars-induced SG formation in JEV-infected cells was due to sequestration of the SG protein Caprin 1 by the viral capsid protein [24]. In the present study, co-localization of WNV capsid with Caprin1 was not observed. In addition, other types of cellular stressors, such as dithiothreitol (DTT) or heat shock, were shown to induce SGs with similar efficiency in mock- and WNV-infected cells indicating that flavivirus-mediated resistance to SG induction is specific for Ars treatment and not due to virus-mediated disabling of SG assembly. Infection of cells with WNV induced the production of detectable ROS and the levels increased with time after infection. However, rapid upregulation of antioxidant pathway gene products mediated by the transcription factors ATF4 and Nrf2 also occurred and increased intracellular GSH levels. The mitochondrial oxidoreductase AIF was also found to contribute to maintaining high GSH levels in WNV-infected cells. The data obtained support the hypothesis that flavivirus infection upregulates both oxidative stress and the antioxidant response in cells creating an altered state characterized by excess antioxidant capacity in infected cells that is sufficient to inhibit SG formation and mitochondrial damage mediated by the ROS induced by the virus infection and Ars-treatment.

## Results

### WNV infection inhibits Ars-induced but not heat shock- or DTT-induced SG formation

**Fig 1:**
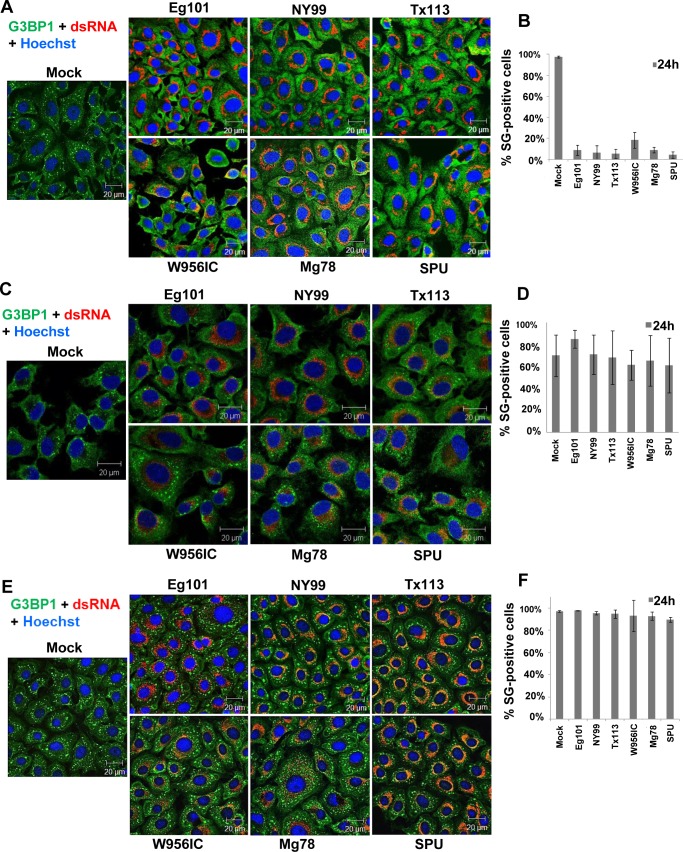
Natural lineage 1 and 2 strains of WNV and ZIKV inhibit Ars-induced but not heat shock- or DTT-induced SG formation. (A) BHK cells were infected with a strain of WNV at a MOI of 1, treated with Ars (0.5 mM) for 30 min at 24 hpi, then fixed, permeablized and analyzed by IFA. SGs were detected with anti-G3BP1 antibody (green) and WNV-infected cells with anti-dsRNA antibody (red). Nuclei were stained with Hoechst 33342 (blue). Images were acquired using a 63X oil immersion objective on a 700 laser scanning confocal microscope (Zeiss). (B) Quantification of mock-infected and WNV-infected cells that were SG-positive after Ars treatment. (C) BHK cells were infected with a strain of WNV at a MOI of 1, treated with 2 mM DTT for 30 min at 24 hpi, then fixed, permeabilized and analyzed by IFA. The images were enlarged slightly to increase visualization of the DTT-induced SGs. (D) Quantification of mock-infected and WNV-infected cells that were SG-positive after DTT treatment. (E) BHK cells were infected with a strain of WNV at a MOI of 1, subjected to heat treatment at 42°C for 30 min at 24 hpi, then fixed, permealized and analyzed by IFA. (F) Quantification of mock-infected and WNV-infected cells that were SG-positive after heat shock. Scale bars, 20 μm.

Treatment of cells with Ars induces oxidative stress that activates HRI kinase to phosphorylate eIF2α leading to SG formation [26, 27]. We previously showed that Ars-induced SG formation was progressively inhibited in WNV Eg101 (lineage 1) and DENV 2 virus infected BHK cells with time after infection [23, 25]. To determine whether this was also a characteristic of infections with additional WNV strains, BHK cultures were infected with a lineage 1 WNV strain (Eg101, NY99, or Tx113), a lineage 2 WNV strain (Mg78 or SPU) or the lineage 2/1 chimeric WNV infectious clone (W956IC) virus at a MOI of 1. At 24 post infection (hpi), cells were treated with 0.5 mM Ars for 30 min and then fixed and processed for immunofluorescence assay (IFA). Consistent with previously reported data [23], ~3–5% of the Ars-treated, WNV Eg101-infected BHK cells contained SGs while ~20% of the Ars-treated, W956IC-infected cells contained SGs (Fig 1A and 1B). W956IC virus-infected cells produce higher levels of viral RNA at early times after infection that is less protected by cell cytoplasmic membranes than the RNA of natural strains of WNV and induce SGs by activating PKR [23]. Similar to what was observed with Ars-treated, WNV Eg101-infected BHK cells, SGs were observed in ~3% of cells infected with each of the other natural WNV strains and treated with Ars for 30 min at 24 hpi (Fig 1A and 1B). In contrast, >90% of the Ars-treated, mock-infected cells contained SGs.

The ability of a WNV infection to inhibit SG assembly induced by activation of PERK was next analyzed. PERK senses accumulation of unfolded proteins in the ER and can be activated by treatment of cells with DTT [28]. BHK cultures were infected with a strain of WNV (MOI of 1), treated with 2 mM DTT for 30 min at 24 hpi, fixed and analyzed by IFA. After DTT treatment, SGs were detected in ~70% or more of mock-infected cells as well as the cells infected with each of the WNV strains tested (Fig 1C and 1D).

Heat shock was previously reported to induce the formation of SGs through activation of HRI or GCN2 [29]. BHK monolayers infected with a strain of WNV (MOI of 1) were incubated at 42°C for 30 min at 24 hpi, fixed and analyzed by IFA. SGs formed in >90% of the mock- and WNV-infected cells in response to heat shock (Fig 1E and 1F). The results indicate that inhibition of Ars-induced SG formation in infected cells is not WNV strain specific and that infections with each of the natural strains of WNV tested can inhibit SG formation induced by Ars but not SG formation induced by other types of stressors. Because no differences were observed in the abilities of the various natural WNV strains tested to inhibit Ars-induced SG formation, WNV Eg101 was used for subsequent experiments.

**Fig 2:**
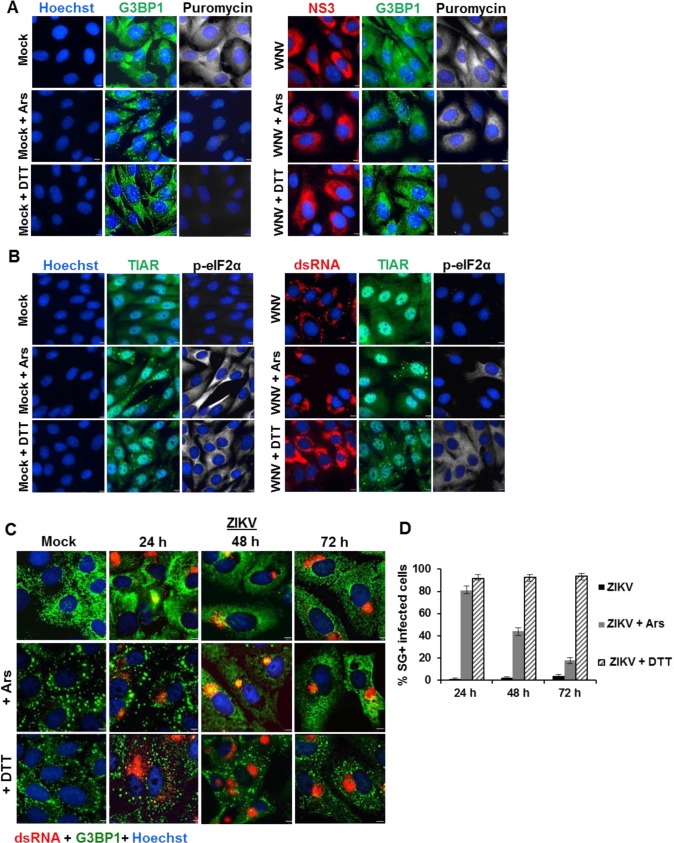
Effect of Ars and DTT on cell translation in WNV-infected cells and analysis of SG induction by DTT and Ars in ZIKV-infected cells. (A) BHK cells were mock-infected or infected with WNV at a MOI 3. At 24 hpi, cells were either untreated or treated with Ars (0.5mM) or 2mM DTT at 37°C for 25 min. Puromycin (50 μg/ml) was then added to the medium in each well for 5 min. The cells were washed twice with PBS and then fixed, permeabilized and processed for IFA. The level of new cell translation was measured with anti-puromycin antibody (white). SGs were detected with anti-G3BP antibody (green) and virus infected cells with anti-NS3 antibody (red). Nuclei were stained with Hoechst 33314 (blue). Cells were visualized with a 100X oil immersion objective on an Axio Observer Z1 wide field fluorescence microscope (Zeiss). Scale bars, 5 μm. (B) BHK cells were infected with WNV at a MOI 3. At 24hpi, cells were either untreated or treated with Ars (0.5 mM) or 2 mM DTT at 37°C for 30 min. Cells were washed, fixed, permeabilized and processed for IFA. The level of eIF2α phosphorylation was assessed with anti-p- eIF2α antibody (white). Anti-G3BP antibody (green) and anti-dsRNA antibody (red). Nuclei were stained with Hoechst 33314 (blue). Cells were visualized with a 100X oil immersion objective on an Axio Observer Z1 wide field fluorescence microscope (Zeiss). Scale bars, 5 μm. (C) Vero cells were infected with a ZIKV FSS13025 at a MOI of 1. At 24, 48 and 72 hpi, cells were untreated or treated with either Ars (0.5 mM) or DTT (2mM) for 30 min and then washed, fixed, permeabilized and analyzed by IFA. SGs were detected with anti-G3BP antibody (green). Infected cells were detected with anti-dsRNA antibody (red). Nuclei were stained with Hoechst 33314 (blue). Cells were visualized with a 100X oil immersion objective on an Axio Observer Z1 wide field fluorescence microscope (Zeiss) and the images were deconvolved. Scale bars, 6 μm. (J) Quantification of SG-positive, ZIKV-infected cells under the conditions tested. A total of 100 cells from each of 2 biological repeats was counted.

Cell translation levels in mock-infected and WNV-infected BHK cells were visualized after treatment with Ars or DTT with a ribopuromycylation assay. Puromycin blocks translation by entering the A-site of ribosomes and is itself transferred to the growing peptide chain, causing the disassembly of polysomes and release of truncated nascent polypeptides containing puromycin instead of normal amino acid at their C-terminus. BHK cells were mock-infected or infected with WNV (MOI of 3), untreated or treated with either Ars or DTT at 24 hpi for 25 min at 37°C. Puromycin (50 μg/ml) was then added to the medium in each well and after 5 min, the cells were washed twice with PBS, fixed, permeabilized and processed for IFA. In mock-infected cells and virus-infected cells, strong cytoplasmic puromycin staining was detected (Fig 2A). After Ars treatment, very little puromycin was detected in mock-infected cells but the level of puromycin detected in WNV-infected, Ars-treated cells was only slightly lower than that in untreated, WNV-infected cells. Very low levels of puromycin were detected in DTT-treated, mock-infected and WNV-infected cells which were all SG-positive cells. The results indicate that the presence of SGs in cells treated with Ars or DTT greatly reduces cell translation levels and that WNV infection inhibits translation reduction by Ars but not by DTT.

SG induction by Ars and DTT is mediated by phosphorylation of eIF2α [5, 6]. BHK cells were mock-infected or infected with WNV (MOI of 3) and untreated or treated with either Ars or DTT at 24 hpi for 30 min. The cells were fixed, permeabilized and processed for IFA. Little eIF2α-phosphorylation was detected at 24 hpi in untreated mock-infected and WNV-infected cells (Fig 2B), consistent with our previously published data [8]. High levels of eIF2α phosphorylation were detected in ~100% of the SG-positive, mock-infected cells after treatment with either Ars or DTT. In contrast, little phosphorylated eIF2α was observed in SG-negative, WNV-infected cells that had been treated with Ars, but high levels were detected in WNV-infected cells after treatment with DTT. These results indicate that WNV-infection inhibits Ars-induced SG formation at a level upstream of eIF2α phosphorylation by the kinase HRI.

We previously showed that DENV2 infection of BHK cells strongly inhibited Ars-induced SG formation by 72 hpi [25]. To determine whether an additional flavivirus, Zika virus (ZIKV) can also inhibit Ars-induced SG formation, Vero cells were mock-infected or infected with ZIKV, strain FSS13025, at a MOI of 1. At 24, 48 and 72 hpi, mock- and virus-infected cells were treated with either Ars (0.5 mM) or DTT (2 mM) for 30 min at 37°C. Cells were then fixed, permeablized and analyzed by IFA. By 72 hpi, similar to mock-infected cells, 3–4% of the infected cells were SG-positive indicating that ZIKV infection does not induce SG formation in Vero cells (Fig 2C and 2D). Either Ars or DTT treatment of mock-infected cells induced SGs in ~100% of the cells. Eighty-one percent of the ZIKV-infected cells were SG-positive after treatment with Ars at 24 hpi, 44% were SG-positive at 48 hpi and 17% were SG-positive at 72 hpi. In contrast, ~90% of the infected cells were SG-positive at each time analyzed following treatment with DTT. The results indicate that ZIKV infections are also able to inhibit Ars-induced but not DTT-induced SG formation but similar to DENV infections, strong inhibition of Ars-induced SG formation is not observed until 72 hpi.

### The cell SG protein Caprin1 is not sequestered by WNV capsid protein in infected BHK cells

**Fig 3:**
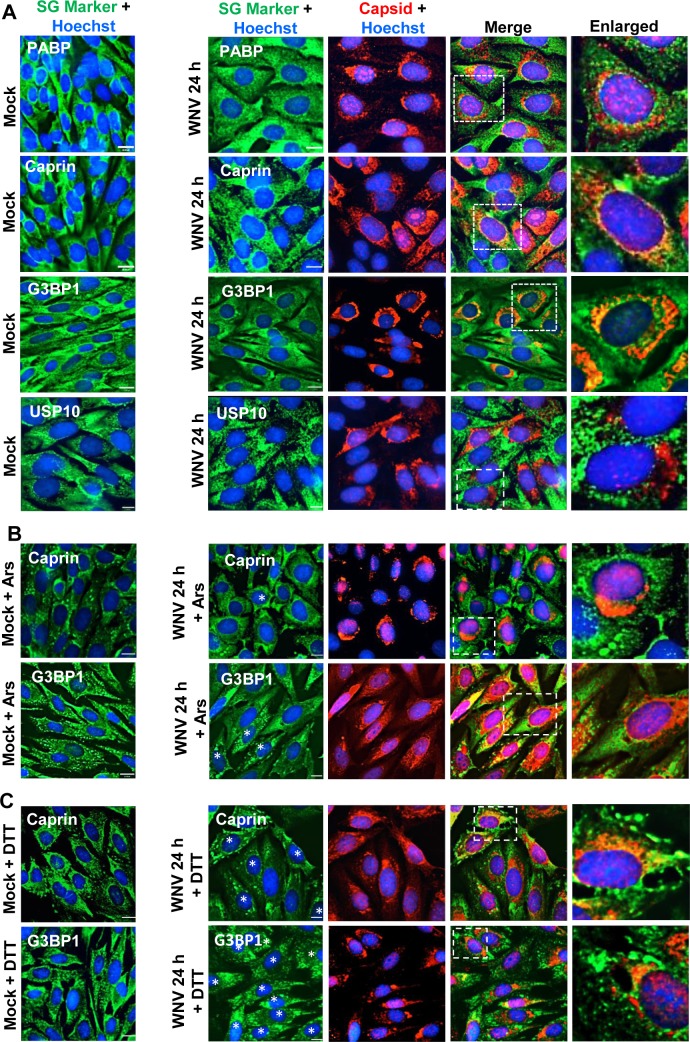
Analysis of the cytoplasmic distribution of WNV capsid protein and various SG proteins in BHK cells. (A) BHK cells were mock-infected (left panels) or infected with WNV Eg101 at an MOI of 3 (right panels). At 24 hpi, cells were fixed, permeabilized and processed for IFA. WNV-infected cells were detected with anti-capsid antibody (red). SG proteins was detected with anti-PABP antibody, anti-Caprin 1, anti-G3BP1 or anti-USP10 antibody (green). (B and C) BHK cells were mock-infected or infected with WNV at an MOI of 3 and at 24 hpi, were treated with either (B) Ars (0.5 mM) or (C) DTT (2 mM) for 30 min and then fixed, permeabilized and processed for IFA. Infected cells were identified with anti-capsid antibody (red) and SGs were detected with anti-caprin 1 or anti-G3BP antibodies (green). Cell nuclei were detected with Hoechst 33342 (blue). Cells were visualized with a 63X oil immersion objective on an Axio Observer Z1 wide field fluorescence microscope (Zeiss) and the images were deconvolved. Scale bars, 11 μm. The image regions indicated by dotted squares were enlarged approximately 2 to 3 times and are shown in the right hand panel of each row. The asterisks indicate infected cells with SGs.

A previous report suggested that suppression of Ars-induced SG formation was the result of sequestration of the SG protein Caprin1 by viral capsid (core) proteins in the cytoplasm of JEV-infected Huh7 cells [24]. Colocalization of Caprin1 with WNV capsid protein was investigated in infected cells. BHK cells were mock-infected or infected with WNV (MOI of 3), untreated or treated with either Ars or DTT for 30 min at 24 hpi, and then fixed and analyzed by IFA. A diffuse distribution throughout the cytoplasm was observed for each of the SG proteins, USP10, PABP, Caprin1 and G3BP1, in mock-infected cells (Fig 3A). Consistent with what was previously shown for flavivirus infections [30], some capsid protein was detected in nuclei while the rest was concentrated in focal areas located primarily in the perinuclear region at 24 hpi with WNV. USP10, PABP, Caprin1 and G3BP1 remained diffusely distributed in the cytoplasm in WNV-infected cells with no obvious enrichment in the focal perinuclear areas containing concentrations of capsid protein. In Ars-treated, mock-infected cells, the majority of Caprin1 and G3BP1 was concentrated in SGs while the reminder was diffusely distributed in the cytoplasm (Fig 3B). In Ars-treated, infected cells without SGs, the capsid protein was located both in the nuclei and in perinuclear focal areas while Caprin1 and G3BP1 remained diffusely distributed in the cytoplasm. In both mock- and WNV-infected cells treated with DTT, SGs formed in the majority of the cells and the majority of the Caprin1 and G3BP1 proteins was located in SGs while the capsid protein was located in perinuclear foci and the nuclei (Fig 3C). The relative amounts WNV capsid protein was similar in untreated, DTT-treated or Ars-treated, infected cells and Caprin1 did not colocalize with WNV capsid protein in either the untreated or treated infected cells. However, WNV infection inhibited Ars-induced SG formation but not DTT- or heat shock-induced SG formation. The results indicate that Ars-induced SG formation is not prevented by sequestration of Caprin1 by the WNV capsid protein.

### WNV infection of BHK cells induces ROS

**Fig 4:**
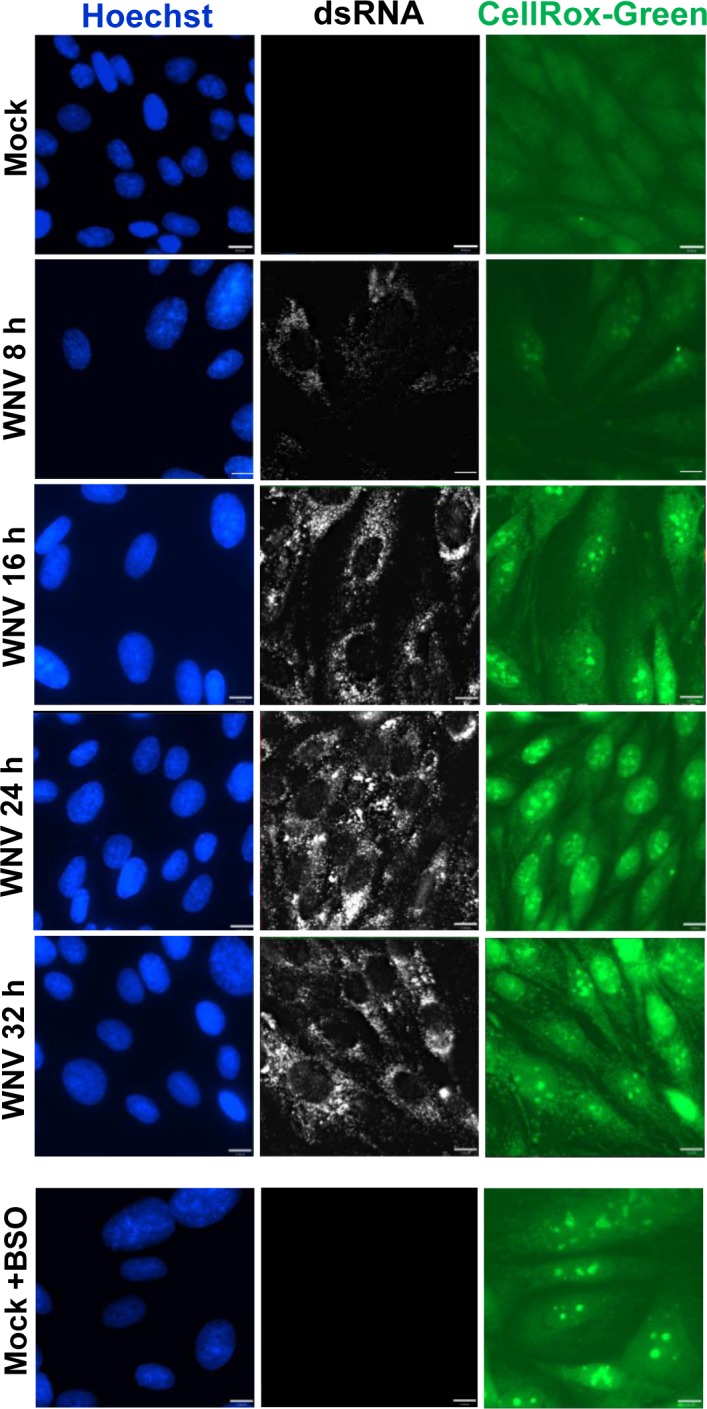
WNV infection induces ROS in BHK cells. BHK cells were mock-infected or infected with WNV Eg101 (MOI of 3) and at 24 hpi, an ROS detection reagent (CellROX Green) was added to the culture media at final concentration of 5 μM. After a 30 min incubation at 37° cells were washed with 1 x PBS, fixed, permeabilized and processed for IFA. WNV-infection was detected with anti-dsRNA antibody (white). Cell nuclei were detected with Hoechst 33342 (blue). Cells were visualized with a 63X oil immersion˚ objective on a widefield fluorescence microscope and the images were deconvolved. Mock-infected cells treated with BSO (1 mM) were used as positive controls. Scale bars, 11 μm.

Flavivirus infections were previously reported to induce ROS production in cells [19–22]. To investigate the generation of ROS in response to WNV infection, cells were mock-infected or infected with WNV (MOI of 3) and incubated with CellROX Green Reagent for 30 min at 8, 16, 24 or 32 hpi. The cells were then washed with phosphate-buffered saline (PBS), fixed and processed for IFA. The intensity of the ROS-specific green signal was low and diffusely distributed in mock-infected cells (Fig 4). By 8 hpi, the signal intensity increased in the cytoplasm of infected cells and some focal nuclear staining was observed consistent with ROS-mediated oxidation of the CellROX Green Reagent and subsequent binding of the oxidized reagent to DNA. By 16 hpi, increased signal intensity in the cytoplasm and brighter nuclear staining were detected in infected cells, and this staining pattern was observed through 32 hpi, indicating that WNV infection induces early and sustained generation of ROS. As a positive control, mock-infected cells were treated with buthionine sulfoximine (BSO). BSO induces oxidative stress in cells by decreasing GSH levels through inhibiting the activity of γ-glutamylcysteine synthetase, an enzyme in the GSH synthesis pathway [31]. Mock-infected cells were treated with BSO (1 mM) for 23 h, incubated with CellRox-Green reagent for 30 min, washed, fixed and processed for IFA. As expected, BSO induced oxidative stress as indicated by an increase in CellRox green intensity compared to that in untreated, mock-infected cells (Fig 4).

### Intracellular GSH levels increase with time after WNV infection

**Fig 5:**
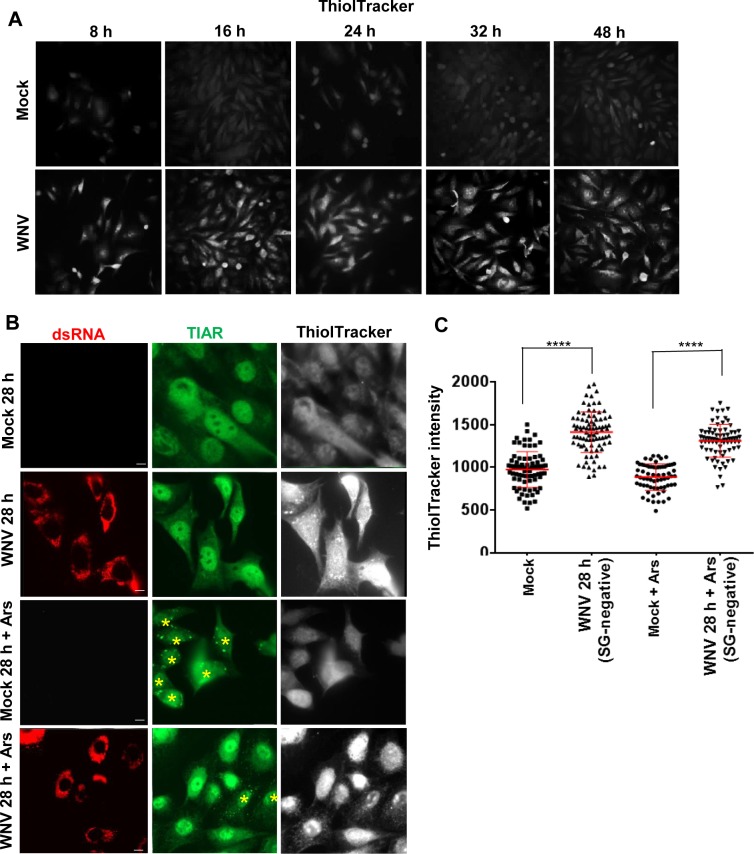
Reduced glutathione (GSH) levels increase with time after WNV infection. (A) Live mock-infected and WNV-infected (MOI of 3) BHK cells were incubated with ThiolTracker Violet (blue) at a final concentration 20 μM in PBS for 30 min at different times after infection and then fixed and immediately visualized under a widefield fluorescence microscope using a 40X objective. (B) Mock-infected and WNV-infected (MOI 3) cells were incubated with ThiolTracker Violet (20 μM) for 30 min at 28 hpi. Replicate cultures were also treated with Ars (0.5 mM) for 20 min at 28 hpi and then the media was replaced with media containing ThiolTracker Violet (20 μM). After another 30 min, the cells were washed, fixed, permeabilized and then processed for IFA. WNV-infected cells were identified with anti-dsRNA antibody (white) and SGs were detected with anti-TIAR antibody (green). Scale bar, 11 μm. (C) The intensity of the ThiolTracker Violet signal in individual cells was quantified using Velocity software. The intensity data were plotted using GraphPad Prism 6. Symbols represent individual cell values. All cells in a category in two fields obtained from each of three biological repeats (total of 6) were analyzed. Longer horizontal lines indicate mean values. Error bars indicate standard deviation (SD). **** P <0.0001.

GSH is the most abundant intracellular antioxidant molecule and protects cells from endogenous and exogenous oxidative stress by donating electrons to ROS [10]. ThiolTracker Violet dye detects intracellular GSH. BHK cells were either mock-infected or infected with WNV at a MOI of 3. At various times after WNV infection, cells were incubated with ThiolTracker Violet for 30 min, washed, fixed and visualized. By 8 hpi, GSH levels were increased in infected cells compared to mock-infected cells (Fig 5A). GSH levels further increased by 16 hpi and remained high throughout the course of the infection. GSH levels were next compared in mock-infected and WNV-infected cells after Ars treatment. Mock-infected or WNV-infected (MOI of 3) BHK cells were incubated with only ThiolTracker or with ThiolTracker Violet and Ars for 30 min starting at 28 hpi and then washed, fixed and analyzed by IFA. After Ars-treatment, ~100% of the mock-infected cells and ~3–5% of the WNV-infected cells were SG-positive. The ThiolTracker signal intensity was consistently higher in WNV-infected, SG-negative cells than in either mock-infected or WNV-infected cells that were SG positive (Fig 5B). The intensity of the ThioTracker signal was quantified in individual cells. The signal intensity was significantly higher in SG-negative, WNV-infected cells than in mock-infected cells. After Ars-treatment, the signal intensity was also significantly higher in SG-negative, WNV-infected cells than in mock-infected cells. The average signal intensity was lower in both mock-infected and WNV-infected cells compared to the respective untreated control cells. The results indicate that GSH levels are increased in WNV-infected cells and are only slightly decreased in these cells after Ars treatment.

### WNV infection upregulates transcription factors that activate antioxidant gene expression

**Fig 6:**
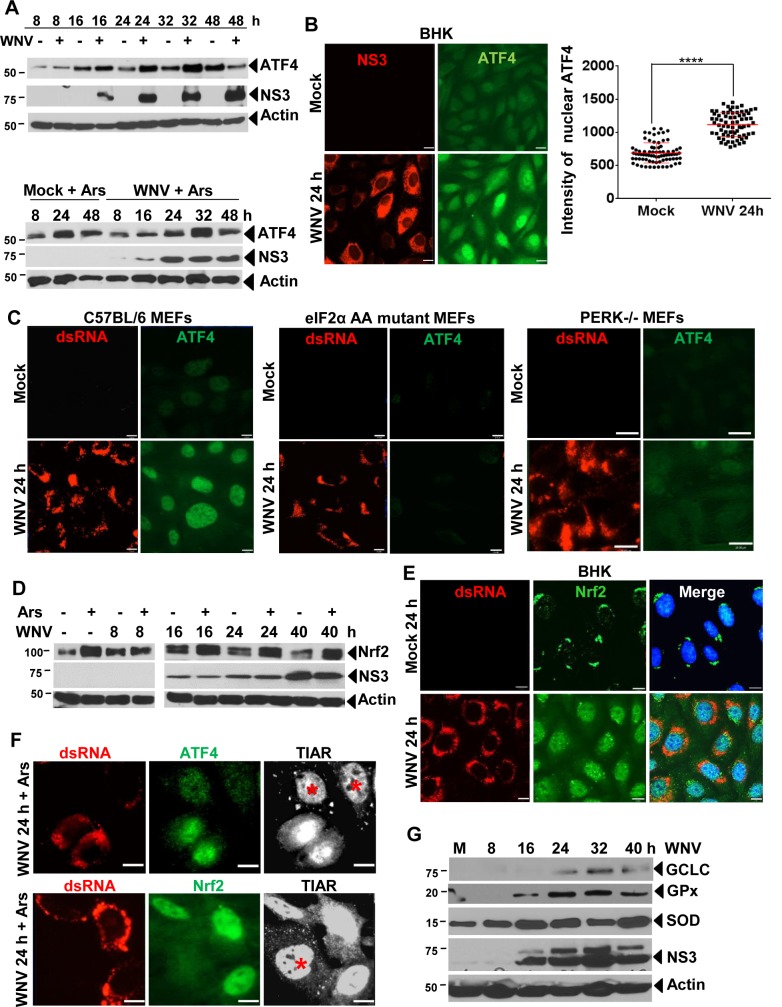
ATF4 and Nrf2 are activated by WNV infection. (A) BHK cells were mock-infected or infected with WNV at an MOI of 1. Some replicate cultures were treated with Ars (0.5 mM) for 30 min at different times after infection and then whole cell lysates were collected in RIPA buffer and analyzed by Western blotting using anti-ATF4 and anti-WNV NS3 antibodies. (B) BHK cells were infected with WNV (MOI of 3) for 24 h. The cells were fixed permeabilized and processed for IFA. The cells were visualized with a 63X oil immersion objective on a widefield fluorescence microscope and the images were deconvolved. Anti-ATF4 antibody (green). Anti-WNV NS3 (red). The fluorescence intensity of nuclear ATF4 in mock-infected and WNV-infected BHK cells was quantified in all cells in a category in two fields obtained from each of three biological repeats (total of 6) using Velocity software. **** P <0.0001. Scale bars, 18 μm. (C) C57BL/6, eIF2α mutant AA, and PERK -/- MEFs were infected with WNV (MOI of 3) for 24 h. The cells were fixed, permeabilized and processed for IFA. Anti-ATF4 antibody (green). Anti-dsRNA antibody (red). Scale bars, 11 μm. (D) Whole cell lysates were harvested from WNV-infected (MOI of 1) BHK cells at different times after infection and used for immunoblotting with anti-Nrf2 antibody. Some replicate cultures were treated with Ars for 30 min at different times after infection. (E) BHK cells were infected with WNV (MOI of 3) for 24 h and the intracellular location of Nrf2 was analyzed by IFA. Images were deconvolved. Anti-p-Nrf2 antibody (green). Anti-dsRNA antibody (red). Nuclei were detected with Hoechst 33342 (blue). Scale bars, 11 μm. (F) Mock-infected BHK cells were treated with Ars (0.5 mM) for 30 min, fixed and processed for IFA. Cells were visualized with a widefield fluorescence microscope and the images were deconvolved. Top row: Anti-dsRNA antibody (red), anti-ATF4 antibody (green), Anti-TIAR (white). Bottom row: Anti-dsRNA antibody (red), anti-Nrf2 antibody (green) and anti-TIAR antibody (white). Scale bars, 18 μm. (G) BHK cells were mock-infected or infected with WNV (MOI of 1) and the whole cell lysates prepared at different times after infection were analyzed by immunoblotting using antibodies specific for the antioxidant enzymes GPx, GCLC and SOD. The asterisks indicate uninfected cells with SGs.

WNV infection increases intracellular GSH levels by 8 hpi (Fig 5A). The transcription factors, NF-E2-related factor 2 (Nrf2) and activating transcription factor 4 (ATF4), activate the expression of many antioxidant pathway genes that produce enzymes involved in GSH synthesis and regeneration [14, 15, 17, 32]. ATF4 protein levels were analyzed by Western blotting of cell lysates harvested at different times after infection of BHK cells with WNV (MOI of 1) (Fig 6A). An increase in the level of ATF4 was observed by 8 hpi with further increases by 16, 24 and 32 hpi. However, the level of ATF4 was consistently reduced at 48 h. Incubation of cells with Ars for 30 min at different times after mock-infection also increased ATF4 levels. ATF4 levels were similar in WNV-infected cells and cells infected with WNV and treated with Ars. ATF4 nuclear translocation was next analyzed by IFA in BHK cells that were mock-infected or infected with WNV (MOI of 3) for 24 h. The ATF4 fluorescence signal was low and diffusely distributed in mock-infected cells (Fig 6B). In WNV-infected cells, the intensity of the ATF4 signal was increased and the ATF4 was observed to concentrate in the nuclei. The intensity of the ATF4 signal was quantified in individual cells at 24 hpi. The average ATF4 signal intensity was significantly higher in WNV-infected cells compared to mock-infected cells. Translation from the far downstream ORF of ATF4 mRNA produces ATF4 protein and requires the presence of phosphorylated eIF2α [33]. Although neither detectable eIF2α phosphorylation [23] nor SG production (Fig 1A) were observed at early times after WNV infection in BHK cells, undetectable transient levels of eIF2α phosphorylation may still have occurred in response to infection [34]. To test this possibility, ATF4 upregulation and nuclear translocation were analyzed by IFA at 24 hpi in control C57BL/6 MEFs and MEFs that express only a non-phosphorylatable eIF2α S51A mutant protein. Upregulation and nuclear translocation of ATF4 were observed in WNV-infected C57BL/6 MEFs but not in eIF2α S51A (AA) MEFs (Fig 6C). Upregulation of ATF4 after WNV infection was also not observed in PERK-/- MEFs. These data indicate that a low level of eIF2α phosphorylation is required for the upregulation of ATF4 expression in WNV-infected cells and that eIF2α phosphorylation is mediated through activation of PERK.

Increased levels of Nrf2 were detected by Western blotting by 8 h after WNV-infection (MOI of 3) and also after Ars-treatment of mock-infected cells for 30 min (Fig 6D). A single Nrf2 band was detected in mock-infected cells but by 16 hpi in WNV-infected cells or after Ars treatment of mock-infected cells, a second band with a higher molecular mass was also detected. Nrf2 was previously shown to be phosphorylated in response to oxidative stress and the size of the additional Nrf2 band detected in WNV-infected cells was consistent with that of phosphorylated Nrf2 [35, 36]. Nuclear translocation of Nrf2 was analyzed by IFA in BHK cells infected with WNV (MOI of 3) for 24 h. Nrf2 was located primarily in the cytoplasm in mock-infected cells (Fig 6E). The intensity of the Nrf2 signal was increased in WNV-infected cells compared to mock-infected cells and the majority of Nrf2 was located in nuclei by 24 hpi. Some replicate infected cultures were also treated with Ars for 30 min at 24 hpi. Consistent with the data in Fig 5B and 5E, bright nuclear signals for both ATF4 and Nrf2 were observed in Ars-treated, WNV-infected cells without SGs (Fig 6F). In SG-positive, un-infected cells that had been treated with Ars for 30 min, some redistribution of ATF4 and Nrf2 occurred but neither protein was strongly concentrated in the nuclei of these cells (Fig 6F). The data indicate that upregulation and nuclear translocation of both ATF4 and Nrf2 occur in response to WNV-infection.

To determine whether ATF4 and Nrf2 nuclear translocation in WNV-infected cells results in antioxidant pathway gene upregulation, the levels of selected antioxidant gene products were analyzed by Western blotting in mock-infected and WNV-infected (MOI of 1) BHK cell lysates at different times after infection. Intracellular levels of superoxide dismutase (SOD) increased by 8 hpi, glutathione peroxidase (GPx) increased by 16 hpi and GCLC increased by 24 hpi (Fig 6G). These data indicate that the expression of antioxidant enzymes involved in the GSH synthesis pathway and GSH regeneration are upregulated in WNV-infected cells.

### ATF4 or Nrf2 knockdown increases the number of SG-positive, WNV-infected cells

**Fig 7:**
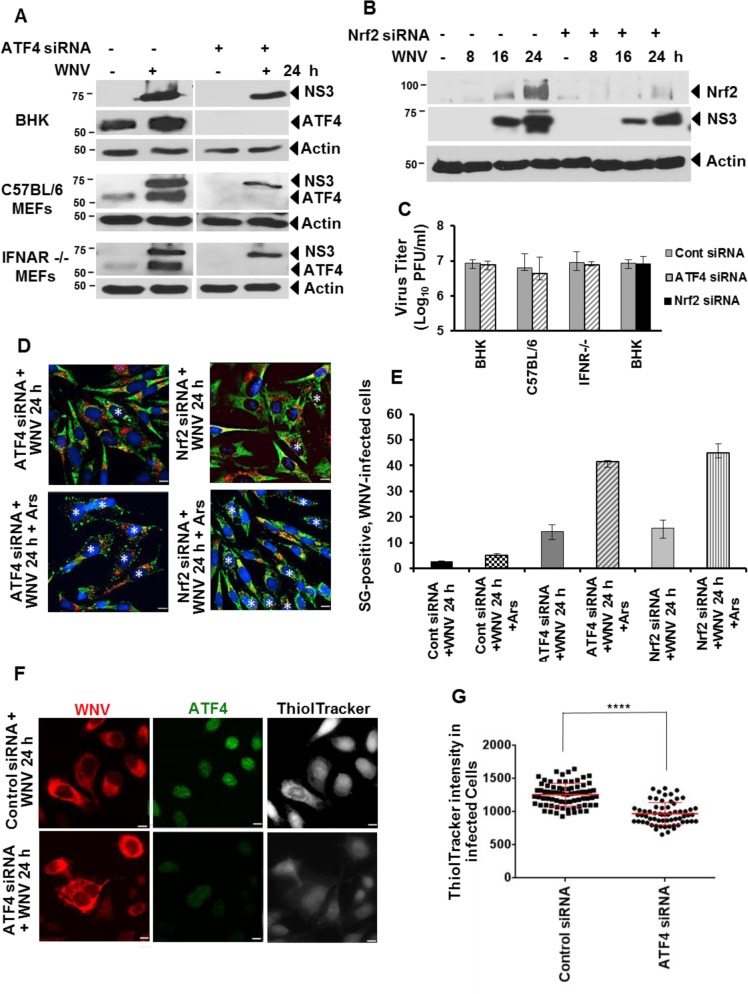
Knockdown of ATF4 or NRF2 increased SG formation in WNV-infected cells. (A and B) BHK and C57BL/6 and IFNAR-/- MEFs cells in wells of a 6-well plate were transfected with 83 pmol/well of ATF4- or Nrf2-specific siRNA or control siRNA for 24 h. The cells were then mock-infected or infected with WNV (MOI of 1). Whole cell lysates was harvested at 24 hpi and analyzed by immunoblotting using anti-ATF4 or anti-NS3 antibody. (C) BHK cells were transfected with control siRNA or specific siRNA and 24 h later infected with WNV (MOI of 1). Culture fluids collected at different times after infection were analyzed for virus infectivity by plaque assay. (D) BHK cells in wells of a 24-well plate were transfected with 17.5 pmol/well of ATF4- or Nrf2-specific siRNA or control siRNA. At 24 hpi, cells were infected with WNV (MOI of 3) for 24 h. Some of the replicate cultures were then incubated with Ars for 30 min, treated and untreated cells were fixed, permeablized and processed for IFA using anti-G3BP antibody (green) and anti-dsRNA antibody (white). WNV-infected, SG-positive cells are indicated by an asterisk. The images were deconvolved. Scale bars, 11 μm. (E) The average number of SG-positive, WNV-infected cells per 100 cells from 2 or 3 fields was determined for each of 3 replicate experiments. (F) BHK cells were transfected with 17.5 pmol/well of ATF4-specific or control siRNA. At 24 h after transfection, cells were infected with WNV (MOI of 3) and 24 h later incubated with ThiolTracker Violet in PBS for 30 min, washed and then fixed, permeabilized and processed for IFA. Anti-WNV antibody (white), anti-ATF4 (red), and ThiolTracker Violet intensity (blue). Scale bars, 11 μm. (G) The ThioTracker signal intensity in individual cells in the different categories in 2 fields from each of three biological replicate experiments (total 6) was quantified using Velocity software. The intensity data were plotted using GraphPad Prism 6. Symbols represent individual cell values. Long horizontal lines indicate mean values. Error bars indicate standard deviation (SD). **** P <0.0001.

The effect of ATF4 or Nrf2 knockdown on WNV replication was first investigated. BHK cells, C57BL/6 MEFs, or IFNAR-/- MEFs were transfected with control or ATF4-specific siRNA. At 24 h after siRNA transfection, cells were either mock-infected or infected with WNV (MOI of 1) and cell lysates harvested at 24 hpi were analyzed by Western blotting. ATF4 levels were efficiently reduced in all three types of cells tested (Fig 7A). Knockdown of ATF4 also resulted in some reduction in viral NS3 levels in all three types of cells (Fig 7A) but only a slight reduction in virus yields was observed with the greatest effect seen in C57BL/6 MEFs (Fig 7C). BHK cells were transfected with control or Nrf2-specific siRNA and then infected with WNV (MOI of 1) 24 h later. Cells lysates were collected at different times after infection and analyzed by Western blotting. Efficient knock down of Nrf2 was observed (Fig 7B). Some reduction in viral NS3 levels (Fig 7B) and a slight reduction in virus yield (Fig 7C) were also detected when Nrf2 was knocked down. A previous study showed that knockdown of Nrf2 in IFN pathway competent cells increased the innate antiviral response [19]. This may explain the negative effect on virus titer observed in C57BL/6 MEFs.

Whether ATF4- or Nrf2-mediated upregulation of the antioxidant pathway is involved in inhibition of Ars-induced SG formation in WNV-infected cells was next analyzed in siRNA transfected BHK cells. Cells were transfected with ATF4- or Nrf2-specific siRNA and 24 h later, the cells were mock-infected or infected with WNV (MOI of 3). At 24 hpi, some of the replicate cultures were incubated with Ars for 30 min prior to fixation and analysis by IFA. After transfection with either ATF4 or Nrf2 siRNA, the IFA signal intensity for the targeted protein was greatly reduced in the majority of the cells (S1 Fig). In either ATF4 or Nrf2 siRNA-transfected cells, the number of SG-positive cells increased after WNV-infection and the number of SG-positive cells further increased with both WNV-infection and Ars-treatment (Fig 7D). The percent of SG-positive, WNV-infected cells was quantified. Transfection of control siRNA did not increase the percent of either WNV-infected or Ars-treated, WNV-infected cells that were SG-positive above that observed in the absence of transfection (~3% of WNV-infected cells and ~5% of WNV-infected, arsenite-treated cells) (Fig 7E). Transfection of ATF4 siRNA increased the percent of SG-positive cells to 14% in WNV-infected cultures and to 42% in WNV-infected, Ars-treated cultures. Transfection of Nrf2 siRNA increased the percent of SG-positive cells to 16% in WNV-infected cultures and to 45% in WNV-infected, Ars-treated cultures. The effect of ATF4 siRNA knockdown on GSH levels in individual WNV-infected cells was analyzed by quantifying ThiolTracker Violet intensity. The average intensity of the ThiolTracker Violet signal was consistently lower in infected cells transfected with ATF4 siRNA than in cells transfected with control siRNA (Fig 7F and 7G). The results indicate that activation of the antioxidant pathway by both ATF4- and Nrf2 in WNV-infected cells contributes to increasing the GSH levels and to inhibiting Ars-induced SG formation.

### Increased GSH levels inhibit Ars-induced SG formation in WNV-infected BHK cells

**Fig 8:**
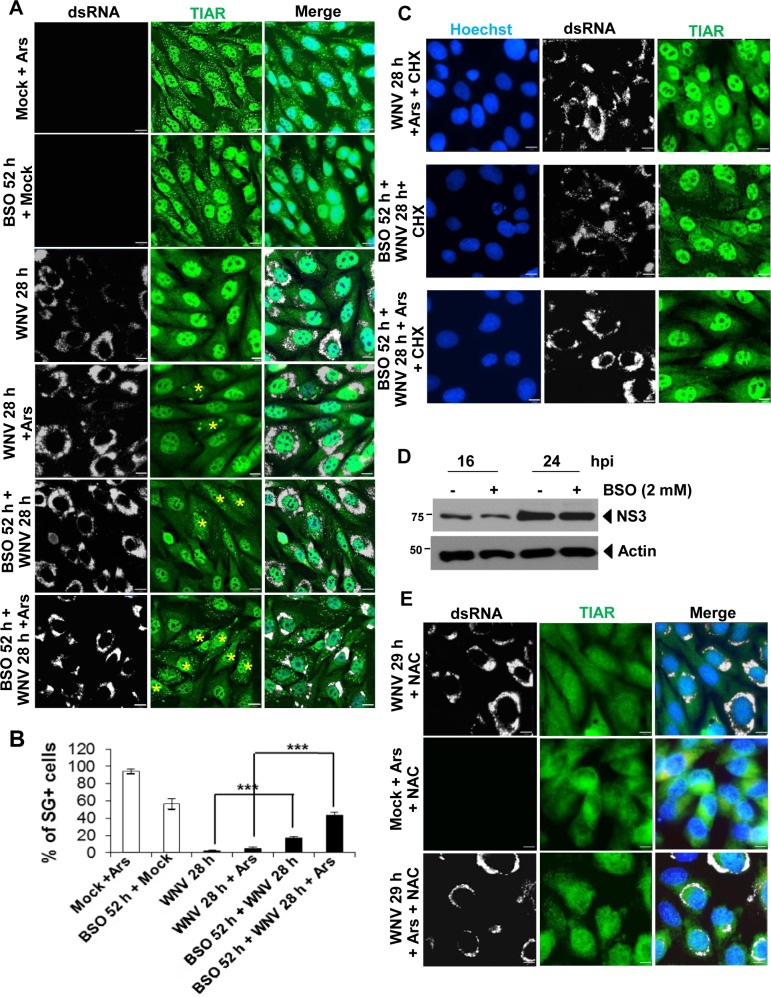
Effect of depleting or supplying GSH on WNV-mediated cell resistance to Ars-induced SG formation. (A) BHK cells at 70% confluency were pretreated with the GSH inhibitor buthionine-sulfoximine (BSO) (2 mM) dissolved in water for 24 h or left untreated. Both types of cultures were then mock-infected or infected with WNV (MOI of 3). BSO (2 mM) was added again to the media of the pretreated cultures after virus adsorption. At 28 hpi, some of the cultures were treated with Ars (0.5 mM) for 30 min and then all of the cells were processed for IFA. Anti-TIAR antibody (green). Anti-dsRNA antibody (white). The images were deconvolved. (B) The number of the SG-positive, mock-infected or WNV-infected cells was determined per 100 cells from 3 to 4 fields for each of 3 biological repeats. Values are averages from two independent experiments. *** P <0.001. (C) Replicate BHK cultures were untreated or pretreated with BSO (2 mM) for 24 h. Cultures were then infected with WNV (MOI of 1) and BSO (2 mM) was again added to the media of the pretreated cultures after virus adsorption. At 28 hpi, Ars (0.5 mM) was added to some of the cultures and 30 min later, cycloheximide (100 μg/ml) was added to all of the cultures for 5 min. Cells were fixed and processed for IFA. Anti-TIAR antibody (green). Anti-dsRNA antibody (white). (D) Lysates harvested from BSO-treated and untreated, WNV-infected BHK cells at different times after infection were analyzed by immunoblotting with anti-WNV-NS3 antibody. (E) BHK cells were mock- or WNV-infected (MOI of 3). At 28 hpi, cells were treated with the GSH precursor NAC (10 mM) with or without Ars (0.5 mM) for 1 h and then fixed and processed for IFA. Anti-TIAR antibody (green). Anti-dsRNA antibody (white). Nuclei were stained with Hoechst 33342 (blue). Scale bars, 11 μm. Images were obtained with a widefield fluorescence microscope and deconvolved.

As another means of investigating whether the increased GSH levels observed in WNV-infected cells were directly involved in inhibiting Ars-induced SG formation, infected cells were treated with BSO to inhibit GSH synthesis. BHK cells were mock-infected or WNV-infected (MOI of 3). Some replicate mock- and WNV-infected cultures were pretreated with BSO (2 mM) for 24 h before infection and then the same concentration of BSO was added to the replacement media after the 1 h virus adsorption period. Cells were harvested at 28 hpi (52 h of BSO treatment). BSO treatment of mock-infected cells induced SG formation in 68% of the cells indicating that BSO-mediated reduction of GSH synthesis in mock-infected cells shifts the redox balance in favor of the ROS which induces oxidative stress and results in SG formation [37] (Fig 8A and 8B). The percent of SG-positive, WNV-infected cells increased from ~3% in untreated cultures to 19.5% after incubation with BSO indicating that BSO-mediated reduction of GSH synthesis reduces the ability of a WNV-infection to counteract the increase in ROS levels induced by the infection.

Additional replicate mock- and WNV-infected cultures were treated only with Ars for 30 min starting at 28 hpi or with BSO before and after infection as described above and then with Ars for 30 min at 28 hpi. As expected due to the increased ROS production induced by Ars, more than 90% of the Ars-treated, mock-infected cells were SG-positive but only about 5% of the WNV-infected cells were SG-positive (Fig 8A and 8B). The percent of SG-positive, WNV-infected cells increased from 19.5% after BSO treatment alone to 41.5% after combined treatment with BSO and Ars. These results indicate that continued GSH synthesis is an important component in determining the amplitude of the virus-induced antioxidant response.

To determine whether the BSO-induced SGs are canonical, cells were incubated with cycloheximide (CHX) for 5 min and then fixed and processed for IFA. CHX efficiently dissolved SGs induced by BSO, as well as those induced by Ars, in both mock-infected cells (S2 Fig) and WNV-infected cells (Fig 8C), strongly suggesting that BSO-induced SGs are canonical and similar to Ars-induced SGs. BSO treatment caused only a slight decrease in intracellular viral NS3 levels (Fig 8D) and had no effect on virus yields (S2 Fig).

It was previously reported that incubation of cells with the GSH precursor N-acetyl cysteine (NAC) protects them from Ars-induced oxidative damage by facilitating an increase in intracellular GSH levels [38]. Cells were treated with NAC as an additional means of analyzing the involvement of increased GSH levels in inhibiting Ars-induced SG formation in infected cells. BHK cells were mock-infected or infected with WNV (MOI of 3) and at 28 hpi, were treated with NAC (10 mM) alone or with both NAC and Ars for 1 h and then fixed and analyzed by IFA. SGs were not observed in any of the Ars-treated, mock-infected cells or Ars-treated, WNV-infected cells that had been treated with NAC (Fig 8E). These data indicate that GSH is directly involved in inhibiting Ars-induced SG formation and strongly suggest that the high intracellular GSH levels induced by WNV-infection play an essential role in inhibiting Ars-induced SG formation in infected cells.

### WNV infection protects both hamster and human cells from mitochondrial damage induced by Ars-induced ROS

**Fig 9:**
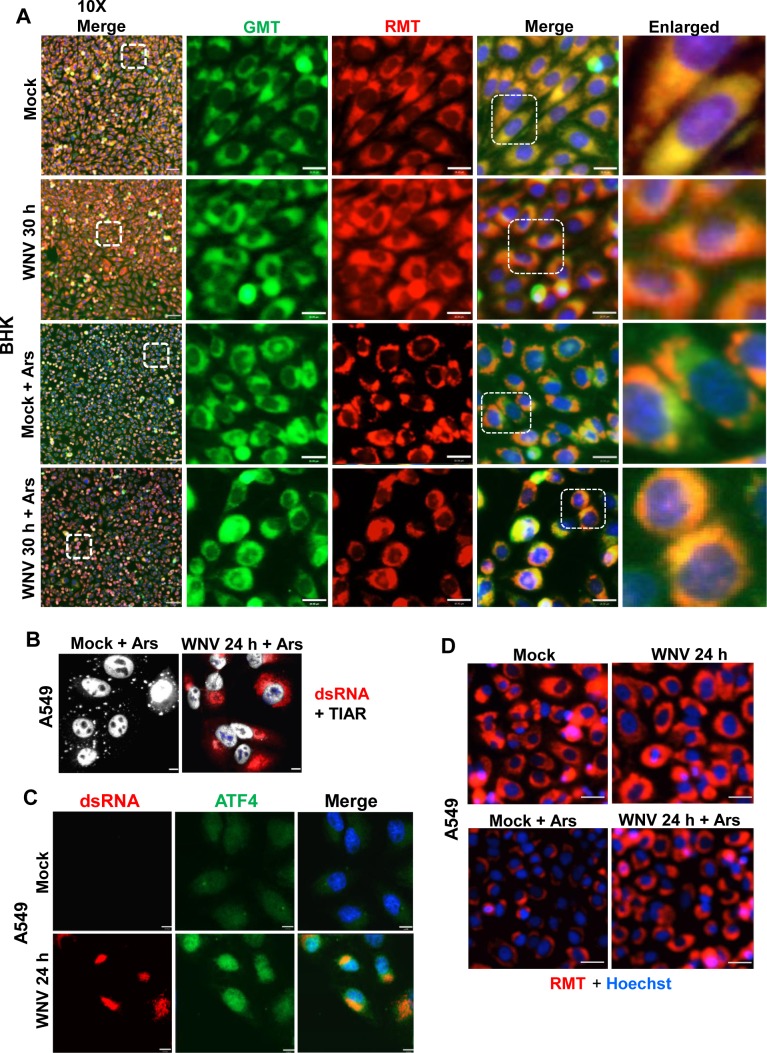
WNV infection protects cells from Ars-induced mitochondrial damage. (A) BHK cells at ~60% confluency in chamber slides were mock-infected or infected with WNV (MOI of 5). At 30 hpi, mitochondria in live cells were stained with both GMT and RMT for 30 min. Ars (0.5 mM) was also added to some wells during the staining period. Hoechst 33342 (blue) was then added to stain nuclei. Live cells were observed directly with a widefield fluorescence microscope using a 10X objective. Areas in the white boxes were enlarged. Scale bars, Left row: 70 μm (10X images) and 18 μm for the first set of enlarged images. (B) Human A549 cells were mock-infected or infected with WNV at a MOI of 3, treated with Ars for 30 min at 24 hpi and then fixed, permeabilized and processed for IFA. Anti-TIAR antibody (white). Anti-dsRNA antibody (red). Scale bars, 11 μm. (C) A549 cells were infected with WNV (MOI of 3) for 24 h and then fixed, permeabilized and processed for IFA. Anti-ATF4 antibody (green). Anti-dsRNA antibody (red). Scale bars, 11 μm. (D) The experiment described in (A) was repeated with A549 cells using only RMT. Scale bars, 18 μm.

In mammalian cells, ROS are generated as normal by-products of mitochondrial respiration [39] but are also produced in response to various environmental stimuli [40]. At low concentrations, ROS are important cellular signaling molecules but at higher concentrations in the absence of an adequate balancing antioxidant response, they cause oxidative stress and mitochondrial damage [41]. As an initial means of comparing the effects on mitochondria of Ars-induced ROS induced in mock-infected and WNV-infected BHK cells, the general condition of the mitochondria was assessed. BHK cells were mock-infected or infected with WNV at a MOI of 5. At 30 hpi, cells were stained with MitoTracker Red CMXRos (RMT) and Mitotracker Green FM (GMT) for 30 min. Live cells were imaged. Ars was also added to some of the replicate cultures at the beginning of the 30 min staining period. RMT detects only normal undamaged mitochondria while GMT detects the total mitochondrial mass [42]. Areas of RMT and GMT colocalization were compared in mock-and WNV-infected cells with and without Ars-treatment. The RMT and GMT signals strongly colocalized throughout the cytoplasm in ~100% of both mock-infected and WNV-infected BHK cells that had not been treated with Ars (Fig 9A). These results indicate that WNV-infection does not induce mitochondrial damage.

In Ars-treated, mock-infected cells, the GMT signal remained distributed throughout the cytoplasm but the RMT signal was detected in only some areas of the cytoplasm consistent with a reduction in the amount of active undamaged mitochondria. In Ars-treated, WNV-infected cells, increased areas of RMT and GMT colocalization were detected in the cytoplasm compared to the Ars-treated, mock-infected cells. The observed increase in RMT-GMT colocalization in WNV-infected, Ars-treated cells indicates that WNV infection inhibits Ars-induced mitochondrial damage.

Inhibition of Ars-induced SG formation and protection of mitochondria from Ars-induced damage by WNV-infection were also studied in the human lung carcinoma cell line A549. A549 cells were either mock infected or infected with WNV at a MOI of 3. At 24 hpi, Ars (0.5mM) was added to the medium and after incubation for 30 min at 37°C, the cells were fixed, permeabilized and analyzed by IFA. Similar to what was observed with Ars-treated, WNV-infected BHK cells, ~3–5% of the Ars-treated, WNV-infected A549 cells were SG-positive and >95% of the Ars-treated, mock-infected cells contained SGs (Fig 9B). Nuclear localization of ATF4 was also analyzed in WNV-infected A549 cells at 24 hpi. The ATF4 fluorescence signal was low and diffusely distributed in mock-infected cells but showed increased intensity and primarily nuclear localization in WNV-infected cells (Fig 9C). These results indicated that WNV infection can activate the anti-oxidant pathway and inhibit Ars-induced SG formation in A549 cells.

To assess Ars-induced mitochondrial damage, A549 cells were mock-infected or infected with WNV at a MOI of 5. At 30 hpi, cells were stained with RMT for 30 min. Ars was also added to some of the replicate cultures at the beginning of the 30 min staining period. Live cells were imaged. The RMT-staining pattern observed in WNV-infected cells.was similar to that in mock-infected A549 cells but the cytoplasmic areas with a detectable RMT signal were much smaller in mock-infected cells after Ars-treatment. In contrast, extensive cytoplasmic areas with RMT signal were detected in the cytoplasm of Ars-treated, WNV-infected cells similar to what was observed in WNV-infected cells. The results indicate that a WNV infection can also protect mitochondria from Ars-induced damage in A549 cells.

### WNV infection induces mitochondrial fusion while Ars-treatment induces mitochondrial fission

Mitochondrial abundance and morphology vary depending on the state of cellular metabolism, the stage of the cell cycle and whether or not cells are undergoing apoptosis [43]. Mitochondria are dynamic and continuously undergoing balanced fusion and fission [44]. Although basal mitochondrial morphology varies among different immortalized cell lines, in the majority, the mitochondria assume either circular or elongated tubular forms. Balanced mitochondrial fission is essential for the generation of additional mitochondria in growing and dividing cells, but elevated levels of ROS cause increased mitochondrial fission. As fission increases, the majority of the mitochondria first became damaged, then severely fragmented and finally aggregate into blob forms [45]. Mitochondrial fusion counteracts stress by allowing functional mitochondria to complement dysfunctional mitochondria through diffusion of internal components resulting in a more interconnected mitochondrial network that enhances communication with the endoplasmic reticulum [44]. In mammals, fusion between the outer membranes of individual mitochondria is mediated by the membrane-anchored dynamin family members Mfn1 and Mfn2, while mitochondrial fission is mediated by the cytosolic dynamin family member Drp1 [46].

**Fig 10:**
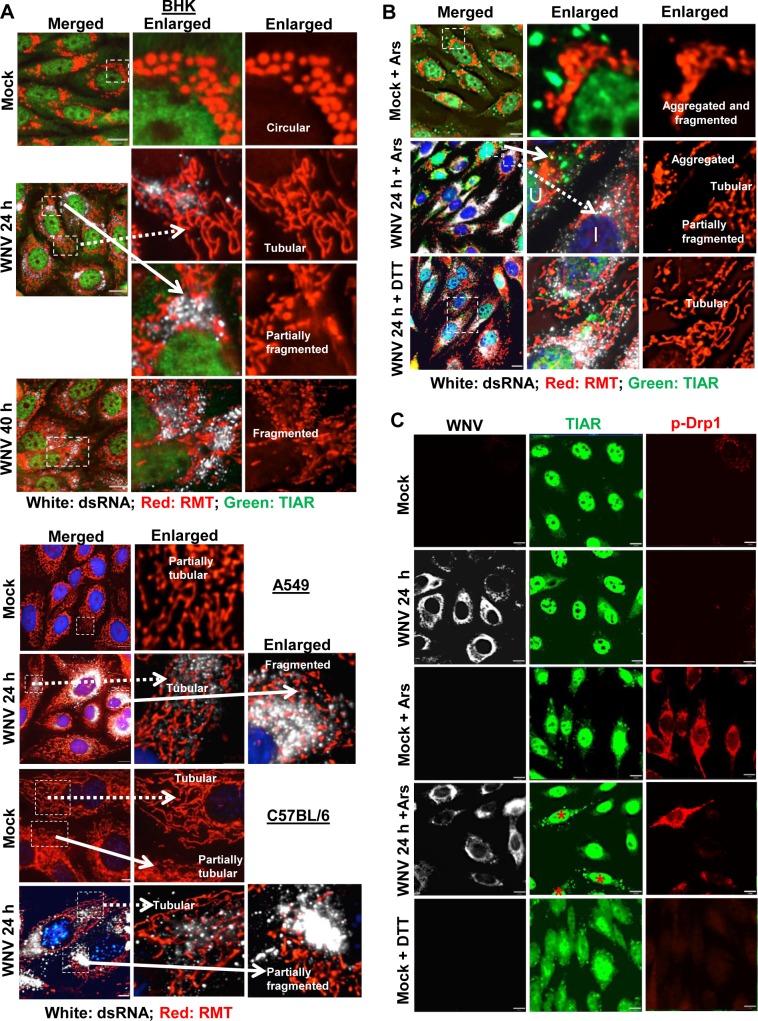
WNV infection induces changes in mitochondrial morphology. (A) BHK cells, C57BL/6 MEFs and A549 cells were mock-infected or infected with WNV (MOI of 3). At 24 h after infection, cells were incubated with RMT for 30 min. Cells were then washed with 1 X PBS, fixed, permeabilized and processed for IFA. Areas in the white boxes were enlarged. Circular, tubular and fragmented mitochondria are indicated. (B) BHK cells were mock-infected or infected with WNV (MOI of 3) and at 24 hpi incubated with RMT for 30 min. Ars (0.5 mM) or DTT (2 mM) was added to some replicate cultures with the RMT. Cells were fixed, permeabilized and processed for IFA. (C) BHK cells were mock-infected or infected with WNV (MOI of 3) and at 24 hpi, Ars (0.5 mM) or DTT (2 mM) was added for 30 min. Cells were fixed, permeabilized and processed for IFA. Anti-dsRNA antibody (white). Anti-TIAR antibody (green). Anti-p-drp1 antibody (red). Cells were visualized with a wide field fluorescence microscope using a 63X objective and the images were deconvolved. Scale bars, 11 μm.

The effects of a WNV infection on mitochondrial morphology were first compared in different cell lines. BHK cells, C57BL/6 (3T3) MEFs and A549 cells were mock-infected or infected with WNV (MOI of 3), incubated with RMT for 30 min at 24 or 40 hpi and then washed in PBS, fixed and analyzed by IFA. In both uninfected (S3 Fig) and mock-infected BHK cells, the majority of the mitochondria were circular (Fig 10A). The morphology of the mitochondria in BHK cells changed with time after infection and by 24 h, the majority of the mitochondria were in an elongated tubular form. However, at both 24 and 40 hpi, focal areas of fragmented or partially fragmented mitochondria were observed adjacent to regions of the ER that contained high concentrations of viral replication complexes. In both uninfected (S3 Fig) and mock-infected A549 cells and C57BL/6 MEFs, the mitochondria were partially tubular/tubular and the morphology did not change after WNV infection except in regions with high concentrations of viral replication complexes where they were partially fragmented (Fig 10A).

The effects of Ars or DTT treatment on mitochondria morphology were next investigated. BHK cells were mock-infected or infected with WNV at a MOI of 3 and at 24 hpi, cells were treated for 30 min with Ars or DTT and RMT prior to fixation and analysis by IFA. In mock-infected cells, the mitochondria morphology changed from circular to highly fragmented and aggregated after treatment with Ars for 30 min, indicating that excessive mitochondrial fission had occurred (Fig 10B). In contrast, the mitochondria in SG-negative, WNV-infected cells treated with Ars at 24 hpi were primarily in an elongated tubular form indicating that WNV infection protects mitochondria from Ars-induced fission [compare the infected (I) and uninfected (U) cells in the enlarged panels of Fig 10B]. As in Fig 10A, focal areas of partial mitochondrial fragmentation were detected in areas with high concentrations of viral replication complexes. When WNV-infected BHK cells were treated with DTT, SG-positive, infected cells contained mostly tubular mitochondria similar to untreated WNV-infected BHK cells consistent with the lack of ROS induction by DTT.

Cyclic AMP-dependent protein kinase phosphorylation of Drp1 at S616 regulates its GTPase activity and increased levels of p-Drp1 are indicative of increased mitochondrial fission [47]. Phosphorylation of Drp1 was investigated in mock-infected and WNV-infected cells with or without Ars-treatment. BHK cells were mock-infected or infected with WNV (MOI of 3). At 24 hpi, some replicate mock- and WNV-infected cultures were treated with Ars for 30 min prior to fixation and analysis by IFA. Low levels of p-Drp1 were detected in some mock- and WNV-infected cells (Fig 10C). After Ars-treatment, the majority of the mock-infected cells contained high levels of p-Drp1 and SGs. In contrast, Ars-treated, WNV-infected cells that were SG-negative contained low levels of p-Drp1 similar to some of the mock-infected cells while the few Ars-treated, WNV-infected cells that were SG-positive contained high levels of p-Drp1. When mock-infected cells were treated with DTT, which does not induce oxidative stress but does induce SG formation, no increase in Drp1 phosphorylation was observed. The results indicate that incubation of mock-infected cells with Ars for 30 min induces extensive Drp1 phosphorylation as well as mitochondrial fragmentation and aggregation while WNV-infection prevents Drp1 phosphorylation and protects mitochondria from Ars-induced fission.

### The mitochondrial protein AIF contributes to suppressing Ars-induced SG formation in WNV-infected cells

**Fig 11:**
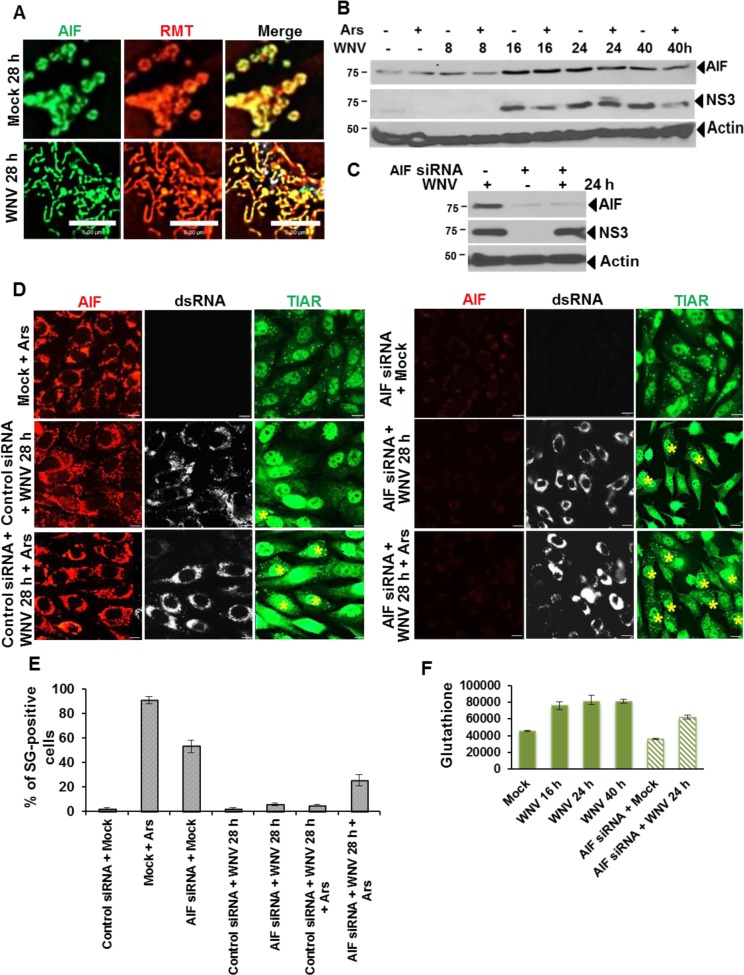
Knock down of AIF increases SG formation in response to Ars. (A) BHK cells were mock-infected or infected with WNV (MOI of 3). At 28 hpi, cells were incubated with RMT for 30 min, fixed and processed for IFA. RMT (red). Anti-AIF antibody (green). Images were acquired with a wide field fluorescence microscope and deconvolved. Scale bars, 6 μm. (B) Cell lysates were prepared from Replicate mock-infected and WNV-infected (MOI 1) cell cultures at different times after infection and analyzed by Western blotting with anti-AIF antibody. Replicate cultures were treated with Ars for 30 min before harvest (C) BHK cells were transfected with AIF siRNA (83 pmoles/well in a 6 well plate). After 24 h, the cells were mock-infected or infected with WNV (MOI 1). At 24 hpi, cell lysates were harvested analyzed by Western blotting. Non-transfected, WNV-infected (MOI 1) whole cell lysates harvested at different times after infection were used as positive controls. (D) BHK cells were transfected with AIF siRNA (17.5 pmoles/well in a 24 well plate) or control siRNA (17.5 pmoles/well). After 24 h, the cells were mock-infected or infected with WNV (MOI 3). At 28 h after infection, cells were treated with Ars for 30 min and then fixed, permeabilized and processed for IFA. Anti-AIF antibody (red). Anti-TIAR antibody (green). Anti-dsRNA antibody (white). Scale bars, 11 μm. (E) The number of SG-positive, mock-infected and SG-positive, WNV-infected cells was determined per 100 cells from 3 to 4 fields for each of three replicate experiments. (F) BHK cells were transfected with 83 pmoles/well of AIF siRNA for 24 h and then infected with WNV (MOI of 1). At 28 hpi, cell lysates were prepared with Apoalert lysis buffer and monochlorobimane was added to a final concentration of 2 mM. The color intensity was measured with a Victor 3 fluorescent plate reader at 395/480 nm. Average values were calculated from 2 wells from each of 2 replicate experiments.

The oxidoreductase activity of the mitochondrial protein AIF is required for maintaining GSH levels during stress [18, 37]. It was previously shown that AIF-deficient cells contain an increased number of SGs after Ars-treatment compared to control cells [37]. The intracellular localization and levels of AIF were analyzed by IFA in BHK cells at different times after mock- or WNV-infection (MOI of 3). As expected, AIF co-localized extensively with RMT in the mitochondria of mock-infected cells and this was also the case for WNV-infected cells (Fig 11A). To determine whether WNV-infection upregulates AIF, cytoplasmic extracts were prepared from replicate mock-infected or WNV-infected (MOI of 1) cultures at different times after infection and analyzed by Western blotting. Some of the replicate cultures were incubated with Ars for 30 min prior to harvest. AIF levels increased by 16 hpi and the increased levels were sustained (Fig 11B). The AIF levels were not further increased by Ars-treatment of infected cells indicating that AIF upregulation is a virus-specific effect.

To determine whether AIF is involved in the inhibition of Ars-induced SG formation in WNV-infected cells, the effect of AIF knockdown was assessed. BHK cells were transfected with either AIF siRNA or control siRNA for 24 h. The cells were then mock- or WNV-infected (MOI of 3). At 28 hpi, some of the replicate cultures were treated with Ars for 30 min and the cells were fixed and analyzed by IFA. AIF knockdown was confirmed by Western blotting of cell lysates by (Fig 11C). In mock-infected cells treated with Ars, AIF displayed a condensed cytoplasmic distribution (Fig 11D) that was consistent with the expected aggregated mitochondrial morphology in these cells (Fig 10B). The cytoplasmic distribution of AIF was more diffuse in WNV-infected cells and Ars-treated, WNV-infected cells without SGs than in Ars-treated, mock-infected cells (Fig 11D) consistent with the morphology of the mitochondria in these cells (Fig 10A and 10B). An increase in the number of Ars-treated, WNV-infected cells with SGs was observed in cultures with AIF knocked down (Fig 11D). The percent of SG-positive cells in cultures with and without AIF knocked down was quantified. AIF knockdown in mock-infected cells increased the percent of SG-positive cells from ~3 to 53.7%. However, AIF knockdown in WNV-infected cultures only slightly increased the percent of WNV-infected, SG-positive cells (from ~3 to 6%). About 5% of the WNV-infected cells were SG-positive after Ars-treatment at 28 hpi but 25.7% of the infected cells were SG positive after AIF knockdown and Ars-treatment (Fig 11E).

The effect of AIF knockdown on GSH levels was next analyzed. BHK cells were transfected with AIF siRNA and 24 h later, were either mock-infected or infected with WNV (MOI of 3). Cells were lysed in ice cold ApoAlert cell lysis buffer and an ApoAlert glutathione detection assay was used to measure intracellular GSH. Consistent with the data shown in Fig 5A, increased GSH levels were detected in WNV-infected cells from 16 through 40 hpi (Fig 11F). AIF knockdown decreased GSH levels in both mock- and WNV-infected cells but the levels of GSH in WNV-infected cells remained higher than those in mock-infected cells.

The data indicate that AIF knockdown in mock-infected cells reduces GSH levels and shifts the redox balance in favor of ROS which induces oxidative stress and SG formation. WNV infection increases GSH levels by almost 2 fold which is sufficient to counteract the increased ROS produced by the virus infection as well as by Ars-treatment so that SGs are not induced. The results indicate that although AIF knockdown reduced the levels of GSH in WNV-infected cells, the GSH levels remained higher than those in mock-infected cells and were sufficient to counteract the increase in ROS produced by the virus infection. However, the GSH levels were sufficient to only partially counteract the ROS induced by Ars-treatment of infected cells. Together the data indicate that AIF upregulation occurs in WNV-infected cells but not Ars-treated cells and AIF plays an important role in maintaining the high levels of GSH in WNV-infected cells required to inhibit Ars-induced SGs.

## Discussion

SGs are dynamic cytoplasmic foci containing aggregated proteins and translationally stalled mRNAs that assemble in cells in response to different cellular stress conditions including virus infection [26]. We previously showed that SGs are not induced by infections with natural strains of WNV in multiple cell lines due to the low levels of RNA synthesis at early stages of the infection cycle and to the association of viral RNA with cytoplasmic membranes, which prevents viral RNA from activating PKR in infected cells [8, 23]. Previous studies showed that WNV-, DENV- and JEV-infected cells are able to inhibit SG formation induced by Ars-treatment [24, 25]. In the present study, ZIKV infection was also shown to inhibit Ars-induced SG formation suggesting that this may be a common characteristic of cells infected with members of the genus *Flavivirus*. Mechanisms used by other types of viruses to counteract SG formation have been investigated. Nsp3 of Old World alpha togaviruses interferes with SG assembly by binding to and sequestering the SG nucleating protein G3BP [48]. The proteases of some picornaviruses and caliciviruses inhibit SG assembly by cleaving G3BP [49–51]. Suppression of SG formation by these viruses extends to stressors that activate any of the four eIF2α kinases because a SG nucleating protein is targeted.

Ars-induced SG formation occurs in response to the production of ROS that activate the eIF2α kinase HRI. Inhibition of SG assembly in Ars-treated, JEV-infected cells was previously reported to be due to the association of viral capsid (core) protein with the SG protein Caprin1 in infected cells [24]. In the present study, Caprin1 was not observed to localize to the focal cytoplasmic areas containing viral capsid protein in WNV-infected cells. Also, although both WNV- and ZIKV-infected cells were resistant to Ars-induced SG-formation, they were not resistant to SG-induction by other stressors such as DTT or heat shock indicating that a step upstream of SG assembly and specific to induction by Ars is inhibited.

Consistent with previous reports that infections with several types of RNA viruses, including WNV, JEV and DENV, induce ROS [19, 20, 52–59], in the present study, WNV-infection was shown to increase ROS levels in BHK cells by 8 hpi. Under basal conditions, low levels of ROS are constantly produced in cells by various metabolic reactions that occur in the mitochondria, the peroxisomes and the ER. The mechanism by which a WNV infection increases ROS production in cells has not yet been determined. However, we previously showed that WNV infection leads to rapid and sustained Ca^2+^ influx during virion endocytosis and through calcium channels in C3H/He MEFs and BHK cells [60]. Increases in cytosolic Ca^2+^ can stimulate mitochondrial ROS production [61]. Viral proteins may also be involved in inducing ROS. The JEV NS5 protein was shown to interact inside mitochondria with the hydroxyacyl-CoA dehydrogenase α and β subunits of the mitochondrial trifunctional protein involved in long chain fatty acid β-oxidation [62]. This interaction impaired fatty acid β-oxidation leading to oxidative stress and the production of increased levels of IL-6 and TNF-α. It has also been suggested that the viral NS2A protein may be involved in inducing oxidative stress in flavivirus-infected cells [22]. The increased ROS levels in flavivirus-infected cells could negatively or positively affect various stages of the virus lifecycle. The efficiency of flavivirus genome capping is increased under conditions of increased ROS [22]. Hsp70, a cellular chaperone protein with important pro-viral functions during multiple phases of the flavivirus lifecycle, is upregulated in response to oxidative stress [63]. In human myeloid-derived dendritic cells, increased ROS elevates the innate immune response to DENV and also leads to activation of bystander cells so that they are less susceptible to viral replication [19].

Treatment of cells with Ars induces high levels of ROS. Cells respond to increased ROS levels by activating endogenous antioxidant defense mechanisms that either scavenge ROS or prevent their formation [61]. Only a few previous studies have investigated the activation of the cellular antioxidant pathways by RNA virus infections. Based on the lack of detectable upregulation of the antioxidant enzyme glutathione S-transferase in DENV-infected BHK cells in one study, it was concluded that flavivirus infection of mammalian cells does not activate the antioxidant pathway. However, strong upregulation of this enzyme was detected in DENV-infected mosquito cells [64]. In other studies, decreased intracellular GSH levels were found in DENV-infected HepG2 cells [65] as well as in other types of cells infected with Sendai virus, influenza virus, or hepatitis C virus [57, 66, 67]. In contrast, in the present study, both a ThioTracker fluorescent assay and a colorimetric assay detected elevated GSH levels in WNV-infected BHK cells by 8 hpi and additional data indicated that multiple antioxidant pathway components were also upregulated by WNV-infection. Similar to our results, a recent study by Olagnier et al [19] showed that DENV infection of human monocyte-derived dendritic cells increased both oxidative stress and upregulated the expression of Nrf2- and Nrf1-mediated antioxidant genes.

The transcription factor Nrf2 regulates inducible expression of genes that have antioxidant response elements (AREs) in their promoters [68, 69] and is the main regulator of the anti-oxidant pathway which controls GSH production and regeneration; GSH utilization; thioredoxin production, regeneration and utilization; and NADPH production. Nrf2 is normally distributed throughout the cytoplasm associated with Kelch-like ECH associated protein 1 (Keap-1) [70]. Ubiquitination of Nrf2 by the KEAP1-Cullin 3 E3 ligase complex results in continuous Nrf2 turnover. Under conditions of oxidative stress, KEAP1 is oxidized [71] and modified at cysteines [72], which releases Nrf2. Phosphorylated Nrf2 translocates to the nucleus. ATF4 is the other primary transcription factor involved in regulating anti-oxidant pathway gene expression. The presence of phosphorylated eIF2α in cells increases ATF4 translation from the downstream ORF on its mRNA [73]. ATF4 can also transactivate gene expression by interacting with various binding partners including Nrf2 [16]. Neither Nrf2−/− nor ATF4−/− MEFs are able to induce the expression of GSH synthesis pathway enzymes [72]. In the present study, siRNA knockdown of ATF4 was found to significantly reduce the upregulation of GSH levels by WNV-infection and siRNA knockdown of either Nrf2 or ATF4 reduced the ability of WNV-infected cells to inhibit Ars-induced SG formation.

Both ATF4 and Nrf2 were rapidly upregulated after WNV infection. It was previously shown that Nrf2 activation in hepatitis C virus-infected cells is mediated by virus-induced Ca^2+^ signaling as well as by elevation of ROS in mitochondria [74]. WNV-infection elevates cytoplasmic Ca^2+^ levels and signaling in C3H/He MEFs and BHK cells [60]. ATF4 levels increased in WNV-infected cells prior to Nrf2 levels. ATF4 translation depends on the presence of phosphorylated eIF2α [33] but phosphorylated eIF2α was not detected in WNV-infected BHK cells by Western blotting. It was previously suggested that levels of phosphorylated eIF2α below the limits of detection by Western blotting could facilitate ATF4 translation in DENV-infected cells [34]. ATF4 activates the expression of Gadd34, a subunit of protein phosphatase 1 (PP1) which dephosphorylates eIF2α and thus, ATF4 negatively feeds back on eIF2α phosphorylation. In the present study, ATF4 upregulation was not observed after WNV infection of MEFs expressing a mutant eIF2α that cannot be phosphorylated or of PERK-/- MEFs indicating that ATF4 upregulation depends on PERK-mediated eIF2α phosphorylation in WNV-infected cells. Although the level of eIF2α phosphorylation induced by WNV-infection is sufficient to activate ATF4 translation, it is not sufficient to induce SG formation. PERK is typically activated by ER stress and the unfolded protein response [75]. The early detection of ATF4 upregulation after WNV infection suggests that PERK is activated by another means than a large accumulation of unfolded viral proteins. Low levels of viral membrane proteins may be able to activate PERK. Expression of WNV NS4A or NS4B alone was previously shown to induce the unfolded protein response and upregulate ATF4 [76].

Mitochondria are continuously undergoing balanced fusion and fission, and this dynamic process is essential for maintaining healthy mitochondrial structure and function. In the present study, the mitochondrial morphology changed from circular to tubular in response to WNV infection in BHK cells but remained tubular in C57BL/6 MEFs and A549 cells. WNV infection also protected mitochondria from Ars-induced fission and damage. Increased levels of p-Drp-1 are indicative of increased mitochondrial fission. The authors of a recent study observed some elongation of tubular mitochondria after infection of Huh7 cells with DENV or ZIKV but not with WNV and equated this with increased mitochondrial fusion [77]. The absence of an increase in mitochondrial fission was attributed to inhibition of Drp1 phosphorylation mediated by the viral protein NS4B and to decreased levels of CDK1, one of the kinases that phosphorylates Drp-1 [46]. In the present study, we also observed no increase in p-Drp1 levels in either WNV-infected cells or in Ars-treated, SG-negative, WNV-infected cells. In contrast, HCV and HBV infections promote mitochondrial fission by inducing the expression and phosphorylation of Drp-1 followed by its translocation from the cytosol to the mitochondria [78, 79]. One study proposed that DENV infections but not JEV infections enhance mitochondrial fission by cleaving the mitochondrial MFN fusion proteins [80] but increased mitochondrial fission was not observed in a subsequent study on DENV infected cells [77].

Although Ars-treatment of BHK cells did not induce upregulation of the mitochondrial protein AIF, we found that AIF was rapidly upregulated by WNV infection and contributed to maintaining high GSH levels in the infected cells. Consistent with these data, a previous study showed that Ars-treatment of AIF-deficient uninfected cells resulted in a marked decrease in both NAD(P)H oxidation and GSH levels and an increase in the number of Ars-induced, SG-positive cells [37].

**Fig 12:**
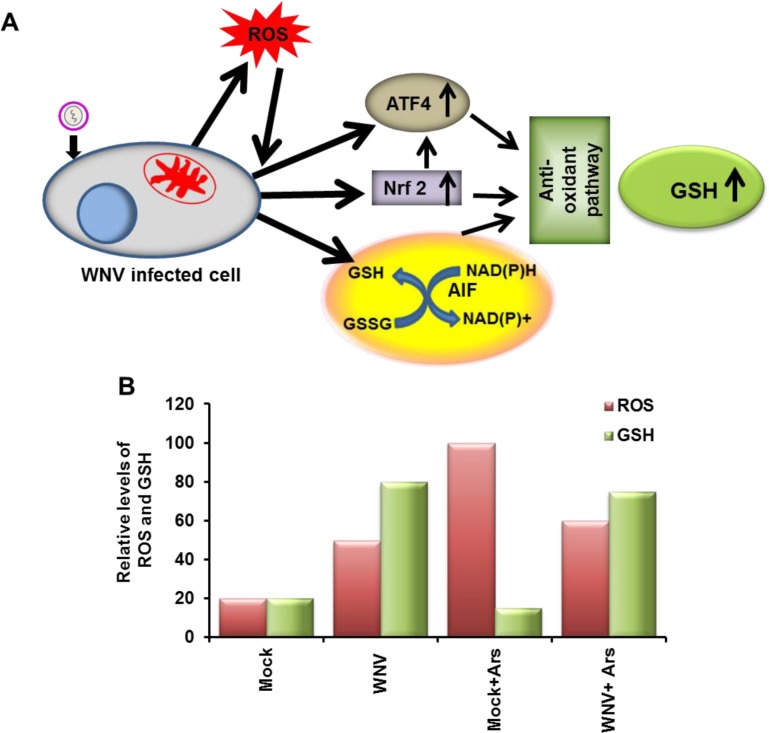
Models of cell redox responses to WNV infection. (A) WNV infection induces the generation of ROS. WNV infection also induces upregulation, activation and nuclear localization of the transcription factors ATF4 and Nrf2, which activate antioxidant pathway genes, as well as upregulation of AIF, which contributes to GSH regeneration from GSSG. (B) Relative levels of ROS and GSH in cells under the different conditions studied. In mock-infected cells, low levels of ROS and GSH are produced and balance each other. Ars-treatment of mock-infected cells rapidly induces high levels of ROS that are much higher than the GSH levels and SGs form. WNV infection induces increased ROS and GSH levels and because the GSH levels are higher than the ROS levels, SGs do not form. Ars-treatment of WNV-infected cells rapidly induces increased ROS levels but the high levels of GSH induced by the virus infection are sufficient to counteract Ars-induced ROS and inhibit SG formation.

In both our previous study [23] and the present study, SG formation in response to Ars-treatment was shown to be inhibited in WNV-infected, BHK cells by 12 hpi. However, the infected cells were not resistant to SG-formation induced by the other types of stressors tested. The data obtained in the present study delineate the mechanism involved in specific inhibition of Ars-induced SG formation by WNV-infected cells. WNV infection induces an increase in intracellular ROS levels by 8 hpi (Fig 12A). The levels of the antioxidant gene transcription factors, ATF4 and Nrf2, are also rapidly upregulated and upregulate the expression of multiple antioxidant pathway enzymes involved in GSH synthesis and regeneration as well as ROS detoxification/neutralization. WNV-infection also rapidly upregulates the levels of the mitochondrial protein AIF. Both the antioxidant pathway genes and AIF contribute to achieving high intracellular levels of GSH in virus-infected cells. Under normal conditions, uninfected cells maintain low and balanced levels of ROS and GSH and SG formation is not induced (Fig 12B). Low levels of ROS have positive functions such as acting as second messengers in a variety of cellular processes including activation of the antioxidant pathway [81]. WNV-infection upregulates ROS levels but also rapidly upregulates the antioxidant pathway gene and AIF levels, which creates a new level of homeostasis with ROS levels higher than those in uninfected cells but counteracted by even higher levels of GSH so that SGs are not induced. Ars-treatment of mock-infected cells rapidly induces high levels of ROS, which reduce GSH levels and cause oxidative stress, mitochondrial damage and finally cell death. Cells respond to the Ars-induced increase in ROS levels by upregulating the expression of genes encoding antioxidant and cytoprotective proteins but this counteraction response is not rapid nor sufficient enough to protect the cells from damage caused by the high levels of ROS that are rapidly induced by Ars-treatment and SGs form. The excess antioxidant capacity of the virus-infected cells is sufficient to balance the ROS induced by both the infection and Ars-treatment, prevent Ars-induced SG formation, protect mitochondria from Ars-induced damage and prolong cell survival. However, the increased antioxidant capacity of WNV-infected cells only provides protection from the effects of stressors that induce oxidative stress and does not inhibit the induction of SGs by other types of stress responses such as those induced by DTT or heat shock.

## Materials and methods

### Cells and viruses

Baby hamster kidney 21, strain WI2 (BHK) cells [82] (obtained from Dr. Tadeusz Wiktor, Wistar Institute, Philadelphia) were maintained in minimum essential medium (MEM) supplemented with 2.5% fetal calf serum (FCS) (obtained from Atlanta Biologicals, Inc.) and 10 μg/ml gentamicin. C57BL/6 MEFs (prepared from pregnant mice originally obtained from Jackson Labs and spontaneously transformed by a 3T3 split schedule) were grown in high glucose Dulbecco's modified Eagle medium (DMEM) supplemented with 10% FCS and penicillin (100 U/ml)/streptomycin (100 μg/ml). Human lung carcinoma A549 cells purchased from ATCC were maintained in F-12K nutrient mixture with (1X) Kaighn modification medium supplemented with 10% FCS and penicillin (100 U/ml)/streptomycin (100 μg/ml). C636 mosquito cells were obtained from ATCC and grown in MEM supplemented with 10% FCS and 1% tryptose phosphate broth and 1% gentamicin. Vero cells were obtained from The Wistar Institute (Philadelphia) and grown in MEM supplemented with 10% FSC and 1% gentamicin.

Homozygous eIF2α S51 (eIf2 A/A) MEF cells (obtained from Dr. Nancy Kedersha, Brigham and Women’s Hospital, Boston, USA) were grown in high glucose DMEM supplemented with 10% FCS, penicillin (100 U/ml)/streptomycin (100 μg/ml),1% pyruvate, 1% glutamine, 1% non-essential amino acids and 2% essential amino acids. PERK^−/−^ MEFs (obtained from Dr. David Ron, University of Cambridge, UK) were grown in high glucose DMEM supplemented with 10% FBS, 1% glutamine, and 10 μg/ml gentamicin. All cells were grown at 37°C in a 5% CO_2_ atmosphere.

A stock of WNV strain Eg101 (obtained from Dr. Hilary Koprowski, Wistar Institute, Philadelphia) was prepared by infecting BHK cell monolayers at a multiplicity of infection (MOI) of 0.1 and harvesting culture fluid at 32 hpi. Clarified culture fluid was aliquoted and stored at -80°C. W956IC virus was prepared as previously described [23]. Aliquots of WNV NY99, Tx113, Mg78 and SPU as well as ZIKV FSS 13025 were provided by Robert Tesh (University of Texas Medical Branch World Reference Center for Emerging Viruses and Arboviruses, Galveston) and a stock of each WNV was grown in BHK cells as described above for WNV Eg101. The titers of the WNV stocks were: Eg101 1x10^8^; W956IC 5x10^7^; NY99 1x10^8^; Tx113 5x10^7^; Mg78 3x10^6^; and SPU 7x10^7^ PFU/ml. Virus infectivity was assessed by plaque assay on BHK monolayers as previously described [83]. A stock of the ZIKV FSS13025 was grown in C636 mosquito cells. Cells were infected at a MOI of 0.1 and media incubated on the infected cells from 48 to 72 hpi was harvested, clarified, aliquoted and stored at -80°C. ZIKV infectivity was assessed by plaque assay on Vero cells using a protocol similar to that for WNV plaque assays on BHK cells. The titer of the stock virus pool was 5X10^6^ PFU/ml.

### Chemicals

Sodium arsenite (Ars), dithiothreitol (DTT) and N-Acetylcysteine (NAC) and puromycin were obtained from Sigma-Aldrich and buthionine sulphoximine (BSO) and cyclyheximide were was obtained from Santa Cruz Biotechnology.

### Fluorescence microscopy, image processing and quantification

BHK cells grown to 50 to 70% confluency on 15-mm diameter glass coverslips in a 24-well plate were infected with WNV at the MOI indicated in Results. For immunofluorescence assays (IFA), cells were fixed with 4% paraformaldehyde in phosphate buffered saline (PBS) for 10 min at room temperature and then permeabilized by incubation with ice-cold 0.1% triton X-100 in phosphate buffered saline (PBS) for 10 min. Coverslips were washed with PBS, blocked for 1 h with 5% horse serum (Invitrogen) in PBS and then incubated with primary antibody diluted in blocking buffer overnight at 4°C. After washing three times with PBS, the cells were incubated with appropriate AlexaFluor secondary antibodies (Invitrogen) (1:400) in blocking buffer. Cell nuclei were stained with Hoechst 33342 dye (Molecular Probes). The coverslips were washed with PBS and mounted on glass slides with Prolong Gold Antifade reagent (Invitrogen). Images were acquired with a 63X oil immersion objective on an LSM 700 laser confocal microscope (Zeiss, Germany) using LSM 5 (version 4.2) software (Carl Zeiss Inc.) or with either a 10X air or 63X or 100X oil-immersion objective on a Zeiss AxioObserver widefield fluorescence microscope and the intensity of fluorescence was analyzed with Velocity software (Perkin Elmer). The number of SG-positive cells per 100 cells were quantified in 3 to 4 fields in 2 to 3 replicate experiments and the average number calculated. Antibodies used for IFA were: mouse anti-dsRNA J2 antibody (English and Scientific Consulting, Hungary) diluted 1:1000 in blocking buffer and rabbit anti-ATF4 (Cell Signaling), goat anti-WNV NS3 (R&D Systems), mouse anti-WNV (MHIAF) (a gift from Robert Tesh, UTMB, Galveston, TX), anti-capsid antibody (a gift from Thomas Hobman, University of Alberta, Edmonton, Canada), rabbit anti-AIF (Cell Signaling Technology), rabbit anti-Nrf2 (Proteintech), rabbit anti-p-Nrf2 (Abcam), rabbit anti-Drp-1 (Cell Signaling Technology), rabbit P-Nrf2 (Abcam), mouse anti-puromycin (Millipore), rabbit p-eIF2α (Cell Signaling), goat anti-TIAR (Santa Cruz Biotechnology), mouse anti-PABP (Thermo Fisher Scientific), rabbit anti-USP10 (Cell Signaling Technology), rabbit anti-G3BP1 (Proteintech), and rabbit anti-Caprin1 (Proteintech) diluted 1:400 in blocking buffer. AlexaFluor donkey anti-mouse or AlexaFluor donkey anti-rabbit or AlexaFluor donkey anti-goat secondary antibodies (Invitrogen) diluted 1:400 in blocking buffer was used.

For live cell imaging, mock-infected or WNV-infected (MOI 5) BHK cells grown to 70% confluency in chamber slides were incubated for 30 min at 37°C with one or more of the staining solutions listed below. CellROX Green reagent (Molecular Probes) was added to the media at a final concentration of 5 μM to detect oxidative stress. ThiolTracker Violet (Molecular Probes) was used at a final concentration 20 μM to detect GSH production. MitoTracker Red-CMXRos (Molecular Probes) and MitoTracker Green were added to media at final concentration of 500 nM to study mitochondrial dynamics. Nuclei were stained with Hoechst 33342 (blue). Mock-infected and WNV-infected cells in some wells were treated with both Ars (0.5 mM) and MitoTracker for 30 min. Cells were observed with a Zeiss AxioObserver widefield fluorescence microscope using a 10X objective.

### Western blot analysis

Confluent monolayers of cells were either mock-infected or infected with WNV at the MOI indicated in Results. Whole cell lysates were prepared at various times after infection as previously described [84] using RIPA buffer containing protease and phosphatase inhibitors (Roche). To prepare mitochondrial extracts, cells were collected by scraping in ice-cold 1X PBS and pelleted at 700 x g for 10 min. The cell pellets were resuspended in ice cold lysis buffer (20 mM HEPES, 10 mM KCl, 1.5 mM MgCl_2_, 0.1% NP-40, 1 mM EDTA, 1 mM DTT, and 250 mM sucrose). After 5 min on ice, the nuclei were pelleted by centrifugation for 10 min at 1000 x g. The supernatant was then centrifuged at 10,000 x g for 20 min and the pellet (mitochondrial fraction) was resuspended in lysis buffer. The supernatant was used as the cytoplasmic fraction. After SDS-PAGE separation, proteins were transferred to a 0.45 μM nitrocellulose membrane (Bio-Rad), that had been blocked with 5% milk in TBST (Tris-buffered saline with 0.5% Tween 20) or 0.5% BSA in TBST for anti-phospho antibodies followed by incubation with primary antibodies. Primary antibodies used were: rabbit anti-ATF4 (Santa Cruz Biotechnology and Cell Signaling), goat anti-WNV NS3 (R&D Systems), rabbit anti-AIF (Cell Signaling), rabbit anti-Nrf2 (Santa Cruz, Proteintech and Cell Signaling), mouse anti-actin antibody (Abcam), rabbit anti-superoxide dismutase (SOD) (Epitomics), rabbit anti-glutathione peroxidase (GPx1) (Epitomics), and rabbit anti-Glutamate cysteine ligase catalytic subunit (GCLC) (Abcam). The secondary antibodies were: anti-rabbit HRP and anti-mouse HRP (Cell Signaling) and anti-goat HRP (Santa Cruz). Blots were treated with chemiluminescence substrate (Supersignal Westpico, ThermoScientific) and the signal was detected using Autoradiography Film (Denville Scientific Inc.).

### Measurement of GSH levels

An ApoAlert Glutathione Detection Kit (Clonetech) was used to measure GSH production in cells. Monolayers of BHK cells in 6-well plates were either mock- or WNV-infected (MOI of 1). At different times after infection, cells from 2 wells (~ 0.5 x10^6^ cell/sample) were collected by scraping in ice-cold 1X PBS and pelleted by centrifugation at 700 x g for 10 min. The supernatant was removed and the cell pellet was washed with ice cold 1 X Cell Wash Buffer (Clonetech). After centrifugation at 700 x g for 30 min, the cell pellet was resuspended in 100 μl of ice-cold 1 X ApoAlert Cell Lysis Buffer (Clonetech), incubated for 10 min on ice, and then centrifuged at maximum speed in an Eppendorf tabletop centrifuge (**~**15,000 rpm) for 10 min. The supernatant was transferred to a new microcentrifuge tube. Monochlorobimane (MCB) was then added (1 μl of 100 mM) to 50 μl of each supernatant in the wells of a 96-well plate. For the negative control, 1 μl of MCB was added to 50 μl of 1 X Cell Lysis Buffer. All samples were incubated at 37°C for 15 min. Fluorescence was measured in a Victor 3 fluorescent plate reader (Perkin Elmer) at 395/480 nm.

### Small interfering RNA (siRNA) transfection

Cells were either seeded in a 6-well or a 24-well plate at ~70% confluency. One hundred pmol CREB-2 (ATF4) siRNA (mouse), Nrf2 siRNA (mouse), AIF siRNA (mouse), or control siRNA (Santa Cruz Biotechnology) were diluted in Opti-MEM and then mixed 1:1 with Lipofectamine RNAiMAX reagent (Invitrogen) diluted 1:16.7 in Opti-MEM. After a 5 min incubation at room temperature, the siRNA-transfection reagent mixture was added to the culture medium. The final siRNA concentrations were 17.5 pmoles in wells of a 24-well plate and 83 pmoles in wells of a 6-well plate. After 24 h of transfection, cells were infected and/or treated with chemicals.

### Statistical analysis

All assays were repeated three times and either average or representative data are shown. Mean and standard deviation (SD) values were calculated using Microsoft Excel or Graph Pad Prism. Significance was determined using an unpaired and two-tailed Student’s t-test.

## Supporting information

S1 Fig Knockdown of ATF4 or Nrf2 in mock-infected cells. BHK cells in the wells of a 24-well plate were transfected with 17.5 pmol/well of either ATF4- or Nrf2-specific siRNA or control siRNA. Twenty four h after transfection, cells were mock-infected. At 24 hpi, cells were fixed, permeablized and processed for IFA using either anti-ATF4 antibody or anti-Nrf2 antibody (green). Nuclei were stained with Hoechst 33342 (blue). Scale bars, 11 μm.
(TIF)

S2 Fig Ars and BSO-induced SGs are dissociated by CHX in mock-infected cells and effect of BSO treatment on WNV yield. (A) BHK cells at 70% confluency were pretreated with BSO (2 mM) for 24 h. Cells were then mock-infected for 1 h and BSO (2 mM) was added to replacement media. Twenty eight h later, some of the cultures were treated with Ars (0.5 mM) and 30 min later, cycloheximide (100 μg/ml) was added to all of the cultures for 5 min. Cells were fixed and processed for IFA. Anti-TIAR antibody (green). Nuclei were stained with Hoechst 33342 (blue). (B) BHK cells were pretreated with BSO (2 mM) or without BSO for 24 h. All of the cultures were then infected with WNV (MOI of 1) and BSO (2 mM) was added again to the media of the BSO-pretreated cultures after the adsorption period. Virus infectivity in media harvested at 16 and 24 hpi was assessed by plaque assay on BHK cells.
(TIF)

S3 Fig Mitochondrial morphology in uninfected cells. BHK cells, C57BL/6 MEFs and A549 cells were seeded on coverslips in a 24 well plate. After 24 h, cells were incubated with RMT (red) and Hoechst 33342 (blue) for 30 min. The cells were then washed with PBS, fixed, and processed for IFA. Cells were visualized with a wide field fluorescence microscope using a 100X objective.
(TIF)
