# Supplementary figures and images for: Arsenite-induced stress granule formation is inhibited by elevated levels of reduced glutathione in West Nile virus-infected cells
Source: PLoS Pathog. 2017 Feb 27;13(2):e1006240. doi: 10.1371/journal.ppat.1006240 (PMC5344523; doi:10.1371/journal.ppat.1006240)

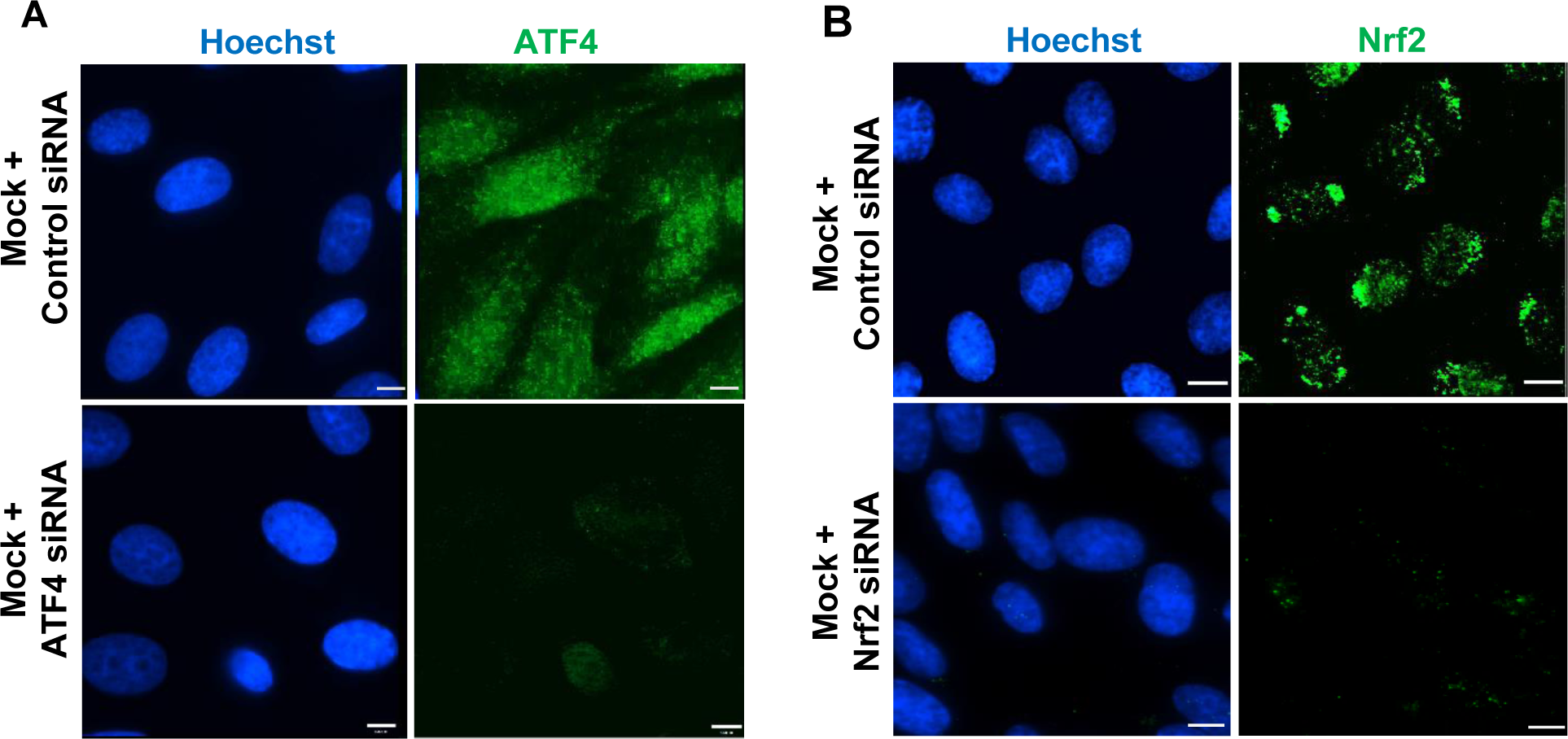

Supplement: S1 Fig — BHK cells in the wells of a 24-well plate were transfected with 17.5 pmol/well of either ATF4- or Nrf2-specific siRNA or control siRNA. Twenty four h after transfection, cells were mock-infected. At 24 hpi, cells were fixed, permeablized and processed for IFA using either anti-ATF4 antibody or anti-Nrf2 antibody (green). Nuclei were stained with Hoechst 33342 (blue). Scale bars, 11 μm. (TIF) [file ppat.1006240.s001.tif]

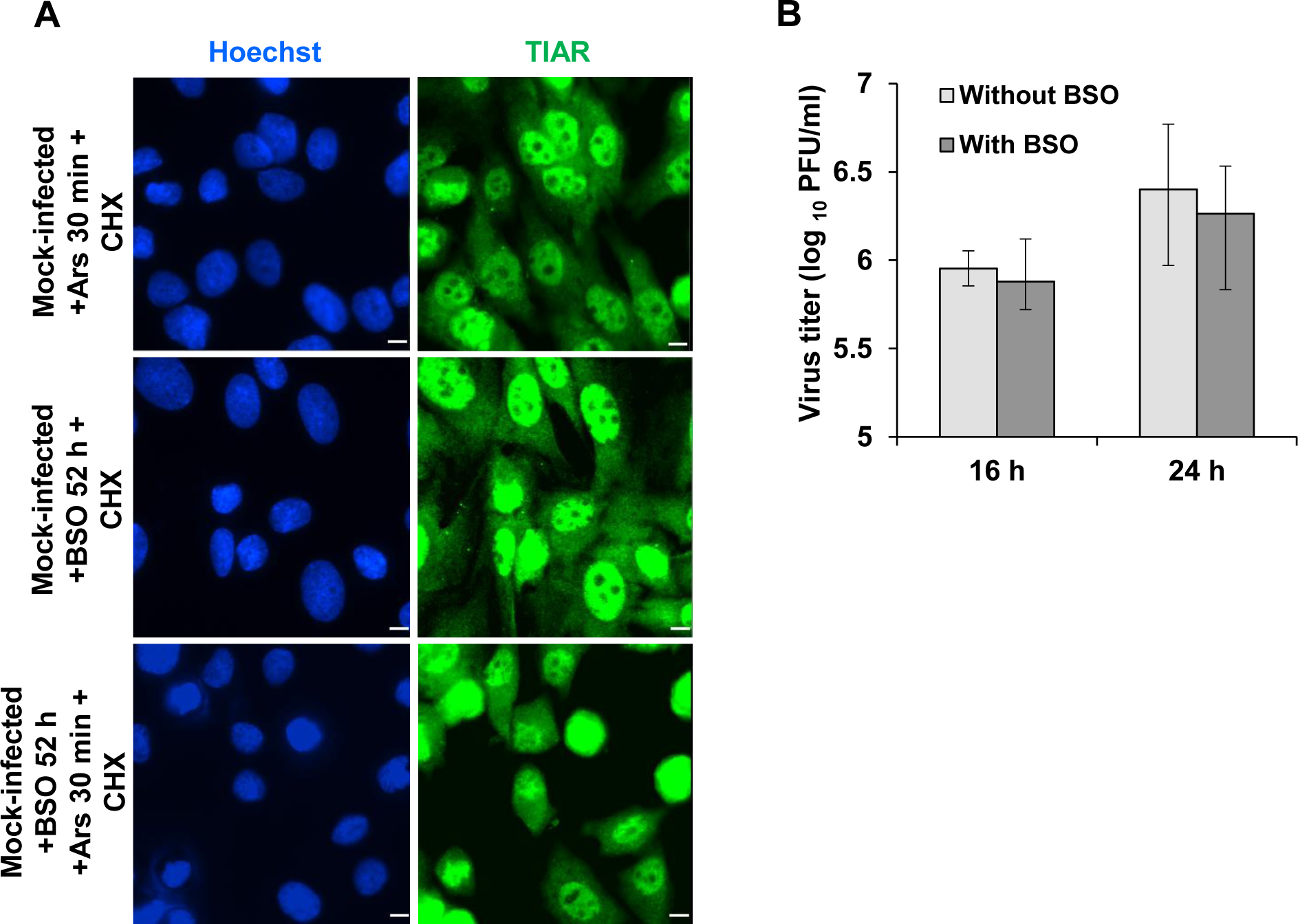

Supplement: S2 Fig — (A) BHK cells at 70% confluency were pretreated with BSO (2 mM) for 24 h. Cells were then mock-infected for 1 h and BSO (2 mM) was added to replacement media. Twenty eight h later, some of the cultures were treated with Ars (0.5 mM) and 30 min later, cycloheximide (100 μg/ml) was added to all of the cultures for 5 min. Cells were fixed and processed for IFA. Anti-TIAR antibody (green). Nuclei were stained with Hoechst 33342 (blue). (B) BHK cells were pretreated with BSO (2 mM) or without BSO for 24 h. All of the cultures were then infected with WNV (MOI of 1) and BSO (2 mM) was added again to the media of the BSO-pretreated cultures after the adsorption period. Virus infectivity in media harvested at 16 and 24 hpi was assessed by plaque assay on BHK cells. (TIF) [file ppat.1006240.s002.tif]

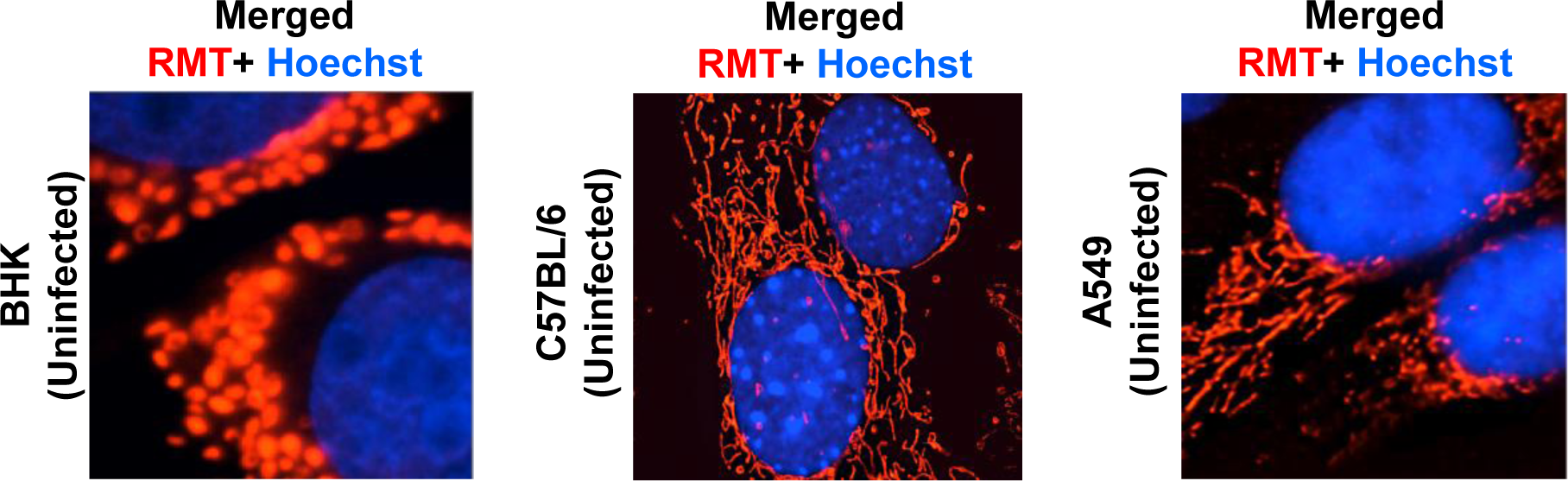

Supplement: S3 Fig — BHK cells, C57BL/6 MEFs and A549 cells were seeded on coverslips in a 24 well plate. After 24 h, cells were incubated with RMT (red) and Hoechst 33342 (blue) for 30 min. The cells were then washed with PBS, fixed, and processed for IFA. Cells were visualized with a wide field fluorescence microscope using a 100X objective. (TIF) [file ppat.1006240.s003.tif]
